# Neuropsychiatric symptoms in preclinical and clinically manifest dementia: clusters and their health determinants

**DOI:** 10.1002/alz.71255

**Published:** 2026-03-08

**Authors:** Anna Giulia Guazzarini, Evangelos Galaris, Virginia Boccardi, Francesca Mancinetti, Carmelinda Ruggiero, J‐Sebastian Muehlboeck, Patrizia Mecocci, Eric Westman, Anna Marseglia

**Affiliations:** ^1^ Division of Gerontology and Geriatrics Department of Medicine and Surgery University of Perugia Perugia Italy; ^2^ Division of Clinical Geriatrics Center for Alzheimer Research Department of Neurobiology Care Sciences and Society, Karolinska Institutet Stockholm Sweden; ^3^ Ageing Epidemiology (AGE) Research Unit School of Public Health, Imperial College London London UK; ^4^ Neuropsychiatric Epidemiology Unit Department of Psychiatry and Neurochemistry Institute of Neuroscience and Physiology, The Sahlgrenska Academy at the University of Gothenburg Mölndal Sweden

**Keywords:** anxiety, clusters, dementia, depression, mild cognitive impairment, neuropsychiatric symptoms

## Abstract

**INTRODUCTION:**

Neuropsychiatric symptoms (NPSs) are common in dementia, but their patterns in preclinical stages remain unclear. This study identified NPS clusters and associated health factors in a geriatric clinical population.

**METHODS:**

We analyzed 1234 participants from the Italian GERIatric COgnitive evaluation memory clinic cohort with Neuropsychiatric Inventory data. Clusters were derived using machine learning (K‐means, Elbow method) separately for dementia and dementia‐free groups. Associations were assessed via multinomial logistic regression.

**RESULTS:**

In the overall cohort, four NPS clusters emerged: minimal NPS, depression‐anxiety‐apathy, depression‐anxiety, and delusions‐agitation‐irritability. Cluster profiles differed between the dementia and dementia‐free groups. Specific clinical and metabolic factors – lipid abnormalities, glycemic control, thyroid dysfunction, and underweight status – were differentially associated with NPS clusters.

**DISCUSSION:**

Distinct NPS patterns exist across the dementia continuum. These clusters differ in demographic, cognitive, functional, and metabolic profiles, suggesting NPS may precede cognitive decline and represent syndromic entities with diagnostic relevance. Multidimensional, personalized approaches are needed.

## Highlights

- Distinct NPS clusters show unique patterns across preclinical and clinical dementia stage.
- NPSs are common in preclinical stages (42%), suggesting behavioral changes may precede cognitive impairment.
- Depression‐anxiety‐apathy clusters span dementia stages, while others are stage‐specific.
- Underweight and hypothyroidism are linked to complex, psychosis‐like NPS clusters.
- Dyslipidemia is a shared feature across clusters; female sex and altered glycemia show stage‐specific associations.

## 1 BACKGROUND

Rising life expectancy and declining fertility rates are driving a global demographic shift toward an aging population, paralleled by a rise in age‐related disorders. Among them, dementia affects 55 million people worldwide and is projected to nearly triple by 2050.^1^

Neuropsychiatric symptoms (NPSs) are highly prevalent across the dementia spectrum, affecting over 90% of individuals throughout the disease course.^2^, ^3^ NPSs may accelerate cognitive progression, worsen quality of life, and contribute to earlier and prolonged institutionalization.^4^ They span multiple domains, including cognitive/perceptual (e.g., hallucinations, delusions), emotional (e.g., depression, anxiety, apathy, irritability), motor (e.g., wandering, aggression), verbal (e.g., screaming, repetitive speech), and vegetative (e.g., sleep and appetite disturbances) symptoms.^5^, ^6^ NPSs are highly heterogeneous, frequently co‐occur, and often cluster into distinct “subsyndromes” such as affective, psychotic, hyperactivity, and apathy, hinting at possibly different underlying mechanisms.^7^ Traditional approaches like factor or principal component analyses capture only some of this complex heterogeneity, leaving the interactions among these factors – and their relationship with dementia progression – poorly understood.^8^ Machine learning‐based clustering allows a more refined characterization of NPSs and offers insights into the neurobiological pathways that may guide future intervention strategies.^9^ Despite its promise, this approach remains relatively underused for identifying NPS patterns in both overt dementia and its preclinical stage. Recent work has applied network analysis to examine symptom‐to‐symptom interactions in patients with dementia and identify central NPSs that may drive co‐occurrence patterns.^10^ While network analysis provides valuable insight into symptom‐level mechanisms, our focus is on person‐level heterogeneity, preferring k‐means clustering, which groups individuals into clinically meaningful profiles based on their overall symptom constellations.

While most research has focused on overt dementia, NPSs may emerge much earlier, potentially decades before clinical symptoms, during the asymptomatic preclinical stage.^11^, ^12^ Approximately 80% of amyloid beta (Aβ)‐positive individuals with subjective cognitive decline have reported NPSs,^11^ and apathy and anxiety have been linked to Aβ accumulation, brain atrophy, and accelerated cognitive decline in dementia‐free older adults.^13^ The NPS profile may differ between preclinical and overt dementia stages. While depression – a core emotional NPS – may be common across both phases,^14^, ^15^ non‐emotional NPSs could manifest earlier, even among cognitively unimpaired individuals. To date, the role of emotional and non‐emotional NPSs in predicting progression of neurocognitive disorders remains largely unexplored.

Several sociodemographic (e.g., age, sex, education, and marital status) and biological factors (e.g., cardiometabolic disorders, inflammation), which are established risk factors for dementia,^16^ have also been linked to NPS onset and severity in mild cognitive impairment (MCI) and early Alzheimer's disease (AD).^17^ However, their impact during the preclinical stage remains unknown, hindering early detection and targeted prevention strategies.

In this study, we aim to identify patterns of NPSs using machine learning‐based clustering in an Italian geriatric population – with and without clinically manifest dementia – and to characterize their associated health‐related factors, including psychosocial and clinical features.

## 2 METHODS

### 2.1 Study population

This cross‐sectional study draws from the GERIatric COgnitive evaluation (GERICO) cohort, an ongoing study of older adults referred to the Memory Clinic at the Geriatric Center for Cognitive Disorders and Dementia (CDCD), within the Gerontology and Geriatrics section of the Department of Medicine and Surgery at the University of Perugia, Italy. Of the 1731 individuals recruited during February 2015 and June 2023, we excluded 483 with missing Neuropsychiatric Inventory (NPI) data and 14 with a diagnosis of epilepsy, resulting in a final sample of 1234 participants with and without dementia for the analysis. Individuals with established primary psychiatric diagnoses (e.g., major depressive disorder, generalized anxiety disorder, bipolar disorder, or psychotic disorders) were not included in this study, as they are typically referred to and treated in specialized units (e.g., psychiatry or psychogeriatrics). Our memory clinic primarily evaluates individuals aged ≥65 years with suspected age‐related cognitive disorders.

Information on sociodemographic and anthropometric factors, medical conditions, medication use, and cognitive functions was assessed through routine clinical examinations conducted by trained nurses, neuropsychologists, and geriatricians. Blood samples were collected after fasting and analyzed at the central laboratory of the University Hospital of Perugia, including panels of biomarkers for lipids, thyroid function, insulin‐related dysregulation (e.g., glucose and glycated hemoglobin [HB1AC]), and vitamins D and B12. More details about the project were previously published.^18^ Data for this study were organized through the HiveDB system.^19^

**RESEARCH IN CONTEXT**

1. **Systematic review**: We reviewed the PubMed literature on NPSs in dementia. Prior studies typically focused on individual symptoms or syndromic combinations. However, this approach may miss patterns not readily visible to the human eye. Data‐driven clustering methods, which can reveal such hidden patterns, have rarely been applied across the full dementia continuum. Also, few studies have examined health‐related determinants of NPS clusters in large, well‐characterized memory clinic cohorts.
2. **Interpretation**: Distinct NPS clusters emerge across the dementia continuum, with some – like depression‐anxiety‐apathy – spanning preclinical and clinical stages, while others are stage‐specific. Their associations with metabolic and endocrine factors, including dyslipidemia, glycemic control, and thyroid function, suggest that NPSs may reflect biologically grounded changes rather than non‐specific behavioral changes.
3. **Future directions**: Longitudinal studies are needed to clarify the temporal relationships between NPSs, metabolic and endocrine dysregulations, and cognitive progression. Integrating neuroimaging, molecular, and lifestyle/risky behavior data may help uncover underlying mechanisms and identify markers for early detection. A better understanding of how NPSs evolve across the dementia continuum could inform stage‐specific prevention and personalized care strategies.

Ethical approval for the GERICO study was granted by CER Umbria – Umbria Regional Ethics Committee protocol number CE‐1065/24 from July 24, 2024. All participants provided written informed consent. The study was performed in accordance with the ethical standards as laid down in the 1964 Declaration of Helsinki and its later amendments or comparable ethical standards.

### 2.2 Clinical assessment of cognitive disorders

A comprehensive neuropsychological assessment was conducted as part of the standard Multidimensional Geriatric Evaluation at the CDCD's Memory Clinic. This evaluation covers routine blood test, physical health, cognition, mental health, functional abilities, social and environmental factors, and supporting clinical diagnosis of cognitive disorders, as well as brain computed tomography (CT) and/or magnetic resonance imaging (MRI) to exclude secondary causes of cognitive disorders.

For the current study, participants were grouped using the Clinical Dementia Rating scale (CDR), with dementia‐free defined as CDR 0 to 0.5 and dementia as CDR ≥1. CDR assesses cognitive and functional decline across multiple domains (memory, orientation, judgment and problem‐solving skills, community relations, home and hobbies, and personal care), with scores ranging from 0 (cognitively unimpaired or subjective cognitive decline), 0.5 (MCI), and 1 to 3 (mild to severe dementia).^20^ These CDR‐based groupings were cross‐checked and confirmed against clinical diagnoses of subjective cognitive decline (SCD), MCI, and dementia made by geriatricians at the CDCD's Memory Clinic as part of routine diagnostic procedures.

SCD was defined as the absence of objective neuropsychological deficits, based on the individuals’ self‐report decline in memory and/or other cognitive abilities relative to their previous level of performance.^21^ MCI was defined as the presence of objective cognitive impairment on neuropsychological testing, without dementia, and with preserved daily functioning.^22^ Dementia diagnoses followed the Diagnostic and Statistical Manual of Mental Disorders–Fifth Edition (DSM‐5) criteria for major neurocognitive disorder,^23^ and subtypes included AD dementia,^24^ vascular dementia (VaD),^25^ and mixed dementia (co‐occurring clinical features of AD and VaD), based on standard diagnostic criteria.

### 2.3 Assessment of NPSs

NPSs were assessed with the NPI,^26^ a structured interview administered to caregivers of individuals with cognitive disorders, designed to assess 12 behavioral symptoms: delusions, hallucinations, agitation/aggression, depression/dysphoria, anxiety, apathy, euphoria, disinhibition, irritability, aberrant motor behavior, night‐time behavioral disturbances/sleep disorders, and appetite/eating disorder. As per NPI scoring, each behavioral symptom was generated by multiplying its frequency (0 “never” to 4 “very frequently, once or more per day, or continuously”) and severity (1 “mild” to 3 “severe”), ranging from 0 to 12, the total NPI score was the sum of the 12 behavioral symptoms (range 0 to 144).

### 2.4 Assessments of health‐related factors

#### 2.4.1 Sociodemographic factors, anthropometrics, and functional status

Education was categorized based on the highest level of formal schooling into low (≤8 years) and high (> 8 years). Marital status was classified into “unmarried,” including single, divorced, or widowed, versus “married/in a relationship,” which included cohabiting. Body mass index (BMI) was calculated as weight divided by height squared (kg/m^2^) and categorized as underweight (<18.5), normal weight (≥18.5 to <25), overweight (≥25 to <30), or obesity (≥30). Daily functioning was assessed using Activities of Daily Living (ADLs)^27^ and Instrumental Activities of Daily Living (IADLs).^28^ ADLs evaluate basic self‐care abilities, including bathing, dressing, eating, toileting, and mobility, with scores ranging from 0 to 6. IADLs measure more complex skills required for independent living, such as managing finances, medication, transportation, and housekeeping, with scores ranging from 0 to 8. Screening for depressive symptoms was assessed using a self‐reported 15‐item Geriatric Depression Scale (GDS‐15) score ≥5.^29^ The Mini‐Mental State Examination (MMSE)^30^ was used to screen for global cognitive impairment.

#### 2.4.2 Chronic medical conditions

Chronic medical conditions were identified based on the International Classification of Disease, 10th revision (ICD‐10)^31^ using data from multiple sources. Medication use was assessed using the Anatomical Therapeutic Chemical (ATC) classification system (available at https://atcddd.fhi.no/atc_ddd_index/). Hypertension was identified based on medical history (ICD‐10 code I10) or use of antihypertensive medication (ATC codes C02, C03, C07, C08, and C09). Diabetes was ascertained based on medical history (ICD‐10 code E11), antidiabetic medication use (ATC code A10), plasma fasting glucose level ≥ 126 mg/dL, or HbA1c ≥ 6.5%.^32^ Additionally, glycemic control was categorized into well‐controlled (HbA1c < 7.5%) and poorly controlled (HbA1c ≥ 7.5%).^32^ Hyperlipidemia was identified based on medical history (ICD‐10 code E78), use of lipid‐lowering medications (ATC code C10), and blood lipid values according to laboratory reference standards at the University Hospital of Perugia. Specifically, altered lipid profiles were defined as “high cholesterol” (total cholesterol ≥ 200 mg/dL), “high triglycerides” (triglycerides > 150 mg/dL), “high LDL” (low‐density lipoprotein) (LDL‐cholesterol [c] > 161 mg/dL), and “low HDL” (high‐density lipoprotein) (HDL‐c < 35 mg/dL in males or < 45 mg/dL in females). Based on these reference values, the parameters were dichotomized accordingly for statistical analysis. Thyroid function was grouped into normal (no medications and thyroid‐stimulating hormone levels ≥0.46 to ≤4.6 mlU/L), hypothyroidism (ATC code H03A or thyroid‐stimulating hormone levels < 0.46 mIU/L), and hyperthyroidism (ATC code H03AB or thyroid‐stimulating hormone levels > 4.6 mIU/L).

A comorbidity index was assessed using the Cumulative Illness Rating Scale for Geriatrics (CIRS‐G),^33^, ^34^ which measures the presence or absence of common aging‐related conditions, consisting of 14 items covering different body systems and their pathologies. The severity of each single item is rated as follows: 1 = no, 2 = mild, 3 = moderate, 4 = severe, 5 = life‐threatening. Also, two summary measures can be constructed: the illness severity index (CIRS‐SI), a summary score based on the average of all CIRS‐G items (total score), excluding psychiatric/behavioral, and the comorbidity index (CIRS‐CI), computed by counting the number of items with a score ≥3 (moderate to severe pathology).^35^

### 2.5 Statistical analysis

#### 2.5.1 Identification of NPS clusters through machine learning

NPS clusters were identified using the k‐means algorithm, an unsupervised machine‐learning method that assigns individual observations to a single cluster based on proximity to a cluster centroid.^36^ We selected k‐means clustering because the method is well suited for grouping individuals based on numeric symptom‐score profiles, providing clear and interpretable centroid‐based symptom patterns that align with our aim of identifying distinct NPS symptom subtypes. The 12 NPSs assessed served as input for the k‐means models. All 12 NPS items were measured on the same 0 to 12 scale, so additional scaling was not applied. Each item, therefore, contributed equally to the distance metric. Four models were trained: (1) using all participants and all NPI item scores; (2) using only participants with a dementia diagnosis and all NPI scores (*n* = 632); (3) using only subjects without dementia and all NPI scores (*n* = 602); and, (4) using all participants and all NPI scores like in Model 1, excluding NPI's depression and anxiety symptoms. The fourth model was developed to identify possible patterns among other NPSs that might be overshadowed by the dominant presence of depression and anxiety symptoms. Each k‐means model was run 100 times with random initializations to ensure stability and avoid dependence on starting centroids. We evaluated cluster solutions with *k* = 2 to 6 clusters; the optimum number of clusters for each model was determined using elbow plots, inertia, and silhouette scores (Figure S1), which identify the point at which adding more clusters minimally reduces within‐cluster variance. Lower inertia values indicate greater within‐cluster compactness, whereas higher silhouette scores reflect better‐defined and more‐separated clusters.^37^

To enhance interpretability, we visualized the symptom profiles of each cluster for all four clustering solutions. For each cluster, we plotted the mean severity of the 12 NPI domains. These profile plots illustrate the dominant symptom and allow comparison of symptom distributions across clusters. Finally, we estimated all mean symptom scores defining each cluster's centroid, representing the multidimensional configuration of NPSs.

To assess the robustness of the clustering results to the ordinal nature of the NPS scores, we conducted sensitivity analyses in which clustering was repeated using k‐medoids with Manhattan and Gower distance metrics. The resulting clustering solutions were compared with the original Euclidean k‐means solution using the Adjusted Rand Index (ARI) (Figure S2). A higher observed concordance indicates that the identified clusters are robust to alternative distance‐metrics that respect score ordinality.

#### 2.5.2 Characterization of NPS clusters

Differences in sociodemographic and health‐related characteristics across clusters were examined using a chi‐squared test for categorical variables, one‐way ANOVA for normally distributed continuous variables, and Kruskal–Wallis test for non‐normally distributed continuous variables. To control for inflated Type I errors due to multiple comparisons, we applied the false discovery rate (FDR) correction across all univariate tests. All reported *p* values are FDR‐corrected, and the significance level was set at 0.05.

For all four clustering models described in Section 2.5.1 – (1) overall sample, (2) dementia group, (3) dementia‐free group, and (4) overall sample excluding anxiety and depression symptoms – multinomial logistic regression models (MLRMs) were estimated. Each model included the same set of predicting factors: sociodemographic (age, sex, education, marital status), functional status (ADL, IADL), clinical conditions (BMI status, hypertension, number of medications, CIRS‐G total score), blood biomarkers (high cholesterol, low HDL, high LDL, high triglycerides, high HbA1C, thyroid function), and cognitive functioning (MMSE). Since all predicting factors were included simultaneously in each model, multiple‐comparisons correction was not applied, consistent with standard statistical practice. Odds ratios (ORs) and 95% confidence intervals (95% CI) were estimated for the associations between cluster allocation (outcome; with the no/minimal NPS cluster as the reference) and potential sociodemographic and health‐related determinants. To assess multicollinearity among predicting factors, we calculated the variance inflation factor (VIF). All variables had VIF values below 5, indicating that multicollinearity wa not a concern.

Statistical significance was defined as *p* < 0.05 for two‐sided tests. Descriptive analyses were conducted using SPSS (version 29.0.1.0), whereas clustering and MLRMs were performed using the “Scikit‐learn” and “Statsmodels” libraries in Python.

## 3 RESULTS

An overview of the health‐related characteristics of the GERICO cohort are summarized in Table S1, whereas the NPSs by dementia status are in Table S2.

**FIGURE 1:**
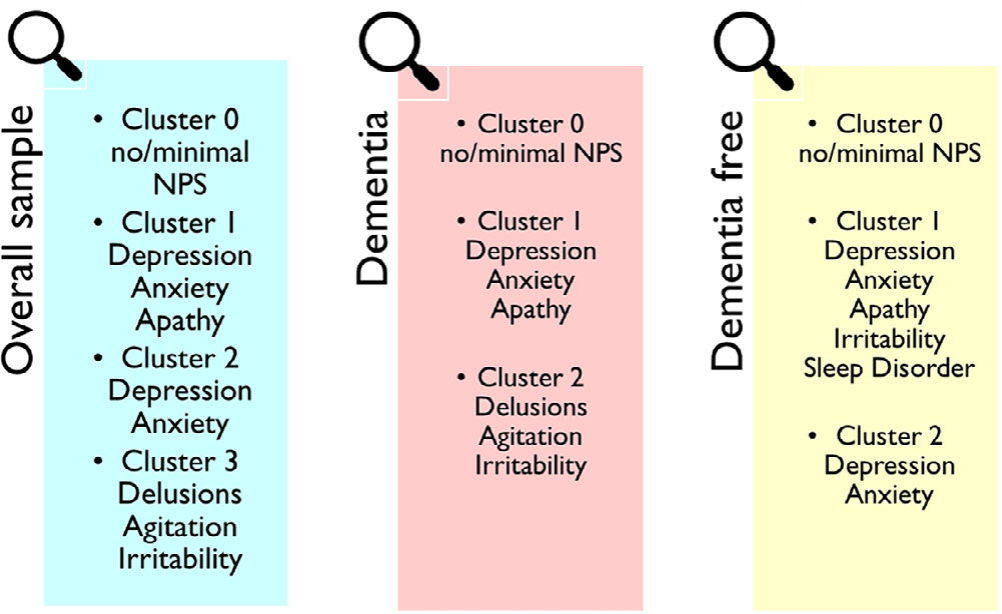
Neuropsychiatric symptom (NPS) clusters identified through machine learning‐based clustering in the overall sample and stratified by dementia status. Three columns represent the distribution of NPS clusters in the overall sample (left, light blue), individuals with clinically manifest dementia (center, pink), and dementia‐free individuals (right, yellow). Cluster 0 consistently reflects no or minimal NPSs across groups. In the dementia group. Cluster 1 is characterized by emotional symptoms (depression, anxiety, apathy), while Cluster 2 includes psychotic and behavioral symptoms (delusions, agitation, irritability). In the dementia‐free group, Cluster 1 includes a broader range of symptoms – emotional, behavioral, and night‐time behavioral, while Cluster 2 is primarily defined by depression and anxiety. These patterns suggest distinct NPS profiles across clinical stages of cognitive decline.

**FIGURE 2:**
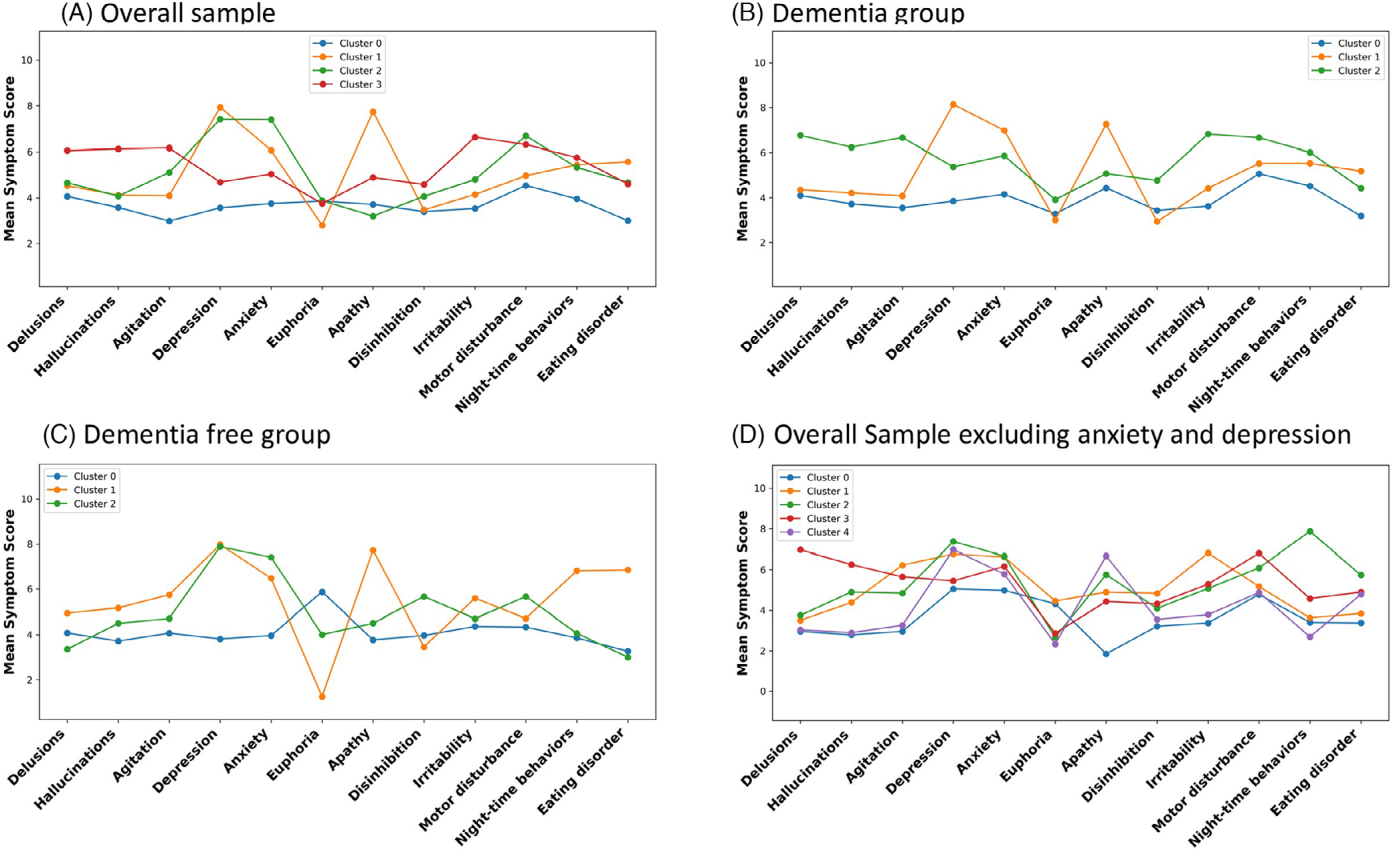
Plots for cluster‐level symptom profiles illustrating mean severity across all 12 NPI domains for each cluster solution. The figure displays four plots illustrate the dominant symptom patterns characterizing each cluster in the overall sample, stratified by dementia status, and in the overall sample excluding anxiety and depression. (A) Overall sample: Cluster 0 with no/minimal NPSs (blue line and nodes); Cluster 1 with depression‐anxiety‐apathy (yellow line and nodes); Cluster 2 with depression‐anxiety (green line and nodes); Cluster 3 with delusions‐agitation‐irritability (red line and nodes). (B) Dementia group: Cluster 0 with no/minimal NPSs (blue line and nodes); Cluster 1 with depression‐anxiety‐apathy (yellow line and nodes); Cluster 2 with delusion‐agitation‐depression‐anxiety‐irritability (green line and nodes). (C) Dementia‐free group: Cluster 0 with no/minimal NPSs (blue line and nodes); Cluster 1 depression‐anxiety‐apathy‐irritability (yellow line and nodes); Cluster 2 depression‐anxiety (green line and nodes). (D) Overall sample excluding anxiety and depression: Cluster 0 with no/minimal NPSs (blue line and nodes); Cluster 1 with agitation‐irritability (yellow line and nodes); Cluster 2 with delusion‐agitation‐irritability (green line and nodes); Cluster 3 with night‐time behavioral disturbances (red line and nodes); Cluster 4 apathy (violet line and nodes).

In the overall sample, the optimal cluster solution revealed four distinct clusters (Figures 1 and 2A). Cluster 0 was the largest (*n* = 557 participants, 45.1%) and reflected no/minimal NPSs, so it was chosen as the reference group. Cluster 1 (*n* = 279, 22.6%) was defined by greater depression and apathy with moderate anxiety. Cluster 2 (*n* = 224, 18.2%) primarily featured high depression and anxiety but minimal apathy. Cluster 3 (*n* = 174, 14.1%) was marked by delusions, agitation, and irritability. Similar patterns were observed in dementia‐free and dementia subgroups, and centroid scores for all NPI domains are reported in Table S3.

Cluster‐specific sociodemographic and clinical features are detailed in Table 1. Cluster 0 (no/minimal NPSs) had the lowest proportions of low education and married or in a relationship, the highest proportion of cognitively unimpaired (*n* = 34, 6.1%) and SCD individuals (*n* = 47, 8.4%), and the fewest comorbidities (median CIRS‐G = 8 [intequartile range {IQR} 6 to 11]). Cluster 1 (depression‐anxiety‐apathy) comprised mostly females (*n* = 153, 68.3%), with the highest prevalence of low education, unmarried, dementia (*n* = 134, 59.8%), and chronic comorbidities (median CIRS‐G = 10 [IQR 7 to 13]). Cluster 2 (depression‐anxiety) had the highest proportion of females (*n* = 219, 78.5%), unmarried, the highest mean MMSE score (23.27 ± 5.23), and the largest representation of MCI (*n* = 127, 45.5%) among clusters. Cluster 3 (delusions‐agitation‐irritability) included the oldest participants (median age 82 [IQR 78 to 85] years), predominantly male, low education, and married/in a relationship. This cluster displayed the most severe NPSs (NPI median score of 30 [IQR 23 to 39]), the lowest median MMSE score (18.78 ± 6.55), the highest prevalence of dementia (*n* = 131, 75.3%), greatest disabilities, and the most severe comorbidities.

**TABLE 1 alz71255-tbl-0001:** Demographic and clinical characteristics by clusters of neuropsychiatric symptoms in GERICO cohort (*n* = 1234).

Characteristics	Cluster 0 (no NPSs) *n* = 557	Cluster 1 (Dep‐Anx‐Apa) *n* = 224	Cluster 2 (Dep‐Anx) *n* = 279	Cluster 3 (Del‐Agi‐Irr) *n* = 174	*p* value^*^
**Demographic**					
Age years	80 (76 to 84)	81 (77.25 to 84.75)	80 (76 to 83)	82 (78 to 85)	<0.001
Female sex	339 (60.9)	153 (68.3)	219 (78.5)	82 (47.1)	<0.001
Education					
Low (≤8 years)	381 (68.4)	175 (78.1)	206 (73.8)	132 (75.9)	0.059
High (>8 years)	176 (31.6)	49 (21.9)	73 (26.2)	42 (24.1)	
Marital status					
Unmarried	146 (41.0)	75 (51.0)	99 (51.3)	46 (42.2)	0.111
Married or in a relationship	210 (59.0)	72 (49.0)	94 (48.7)	63 (54.5)	
BMI	26.55 ± 4.37	26.24 ± 4.51	26.54 ± 4.55	26.51 ± 4.91	0.999
Underweight (<18.5)	9 (1.6)	5 (2.2)	3 (1.1)	8 (4.6)	
Normal (≥18.5 to 25)	167 (30.0)	69 (30.8)	84 (30.1)	41 (23.6)	
Overweight (≥25 to 30)	181 (32.5)	75 (33.5)	89 (31.9)	55 (31.6)	0.567
Obesity (≥30)	97 (17.4)	39 (17.4)	49 (17.6)	28 (16.1)	
ADL score	5 (5 to 6)	5 (3 to 6)	5 (5 to 6)	5 (3‐5)	<0.001
IADL score	4 (2 to 6)	3 (1 to 5)	5 (2.75 to 7)	2 (1 to 4)	<0.001
Depressive symptoms (GDS‐15 score ≥ 5)	162 (31.6)	134 (65.0)	175 (65.3)	52 (38.8)	<0.001
Neuropsychiatric Inventory (NPI) total score	8 (4 to 12)	26 (20 to 36)	21 (16 to 28)	30 (23 to 39)	<0.001
**Cognitive status**					
MMSE score	22.40 ± 5.68	20.65 ± 5.76	23.27 ± 5.23	18.78 ± 6.55	<0.001
Clinical diagnosis					
Unimpaired	34 (6.1)	6 (2.7)	21 (7.5)	5 (2.9)	
SCD	47 (8.4)	5 (2.2)	24 (8.6)	3 (1.7)	
MCI	216 (38.8)	79 (35.3)	127 (45.5)	35 (20.1)	<0.001
Dementia	260 (46.7)	134 (59.8)	107 (38.4)	131 (75.3)	
Cognitive impairment severity. *n* (%)					
CDR = 0 (CU and SCD)	81 (14.5)	11 (4.9)	45 (16.1)	8 (4.6)	
CDR = 0.5 (MCI)	216 (38.8)	79 (35.3)	127 (45.5)	35 (20.1)	
CDR = 1 (mild dementia)	182 (32.7)	82 (36.6)	82 (29.4)	69 (39.7)	<0.001
CDR = 2 (moderate dementia)	67 (12.0)	47 (21.0)	22 (7.9)	46 (26.4)	
CDR = 3 (severe dementia)	11 (2.0)	5 (2.2)	3 (1.1)	16 (9.2)	
**Medical conditions**					
CIRS‐G					
Total score	8 (6 to 11)	10 (7 to 13)	9 (6 to 13)	9 (6.14)	<0.001
Comorbidity index	1 (0‐1)	1 (0‐2)	1 (0 to 1)	1 (0 to 2)	<0.001
Severity index	1.75 (1.5 to 2)	1.88 (1.67 to 2.00)	1.75 (1.40 to 2.00)	2 (1.59 to 2.25)	<0.001
Number of medications	5 (3 to 7)	6 (4 to 8)	5 (4 to 7.75)	6 (4 to 8)	<0.001
Polypharmacy (≥5 drugs)	232 (53.8)	129 (70.5)	143 (62.7)	95 (62.9)	0.003
Stroke	17 (3.1)	8 (3.6)	12 (4.3)	10 (5.7)	0.597
Myocardial infarction	27 (4.8)	7 (3.1)	7 (2.5)	5 (2.9)	0.416
Diabetes	132 (23.7)	54 (24.1)	66 (23.7)	56 (32.2)0	0.232
Cancer	75 (13.5)	40 (17.9)	42 (15.1)	21 (12.1)	0.597
Hyperlipidemia	365 (65.5)	150 (67.0)	196 (70.3)	114 (65.5)	0.667
Hypertension	390 (70.0)	169 (75.4)	203 (72.8)	132 (75.9)	0.463
Thyroid function					
Normal	299 (53.7)	135 (60.3)	153 (54.8)	108 (62.1)	
Hypothyroidism	75 (14.2)	29 (14.7)	42 (17.6)	27 (18.4)	0.980
Hyperthyroidism	36 (6.5)	15 (6.7)	23 (8.2)	13 (7.5)	
**Blood biomarkers**					
Total cholesterol (mg/dL)	196 (170 to 228.75)	188 (163.50 to 222.50)	199 (171 to 227)	183.5(155.25 to 227.75)	0.218
High cholesterol	233 (45.2)	87 (40.8)	123 (46.6)	58 (36.3)	0.232
HDL‐c (mg/dL)	55 (47 to 66)	55 (45 to 65)	55.5 (47 to 67)	51 (43 to 64)	0.057
Low HDL	46 (9.1)	32 (15.2)	33 (12.8)	26 (16.5)	0.064
LDL‐c (mg/dL)	114 (93 to 142)	112.4 (90 to 140)	118.5 (94.5 to 140)	107 (84 to 136.5)	0.295
High LDL	58 (12.6)	21 (10.8)	34 (14.2)	18 (12.8)	0.813
Triglycerides (mg/dL)	107 (80 to 138)	108 (85 to 145.50)	108 (83 to 143)	106 (81 to 147)	0.597
High triglycerides	98 (19.1)	49 (23.0)	58 (22.1)	37 (23.6)	0.608
Glucose (mg/dL)	100 (91 to 113)	100.5 (90 to 116)	99 (89 to 116)	101.5 (93 to 119.25)	0.650
HbA1C %	6.2 (5.7 to 6.9)	6.4 (5.7 to 7.1)	6.4 (5.8 to 07.4)	6.4 (5.7 to 07.2)	0.706
Poor controlled (≥7.5)	16 (14.4)	9 (20.0)	11 (24.4)	8 (22.2)	0.597
Vitamin D (ng/mL)	20.05 (11.52 to 32.55)	24.65 (11.75 to 30.65)	20.9 (12.1 to 030.9)	14.8 (10.7 to 020.3)	0.220
Vitamin B12 (pg/mL)	235 (179 to 331.50)	240 (190 to 330.25)	241 (1800 to 329)	227 (178.50 to 301)	0.672
TSH (µUI/mL)	1.66 (1.10 to 2.43)	1.69 (1.05 to 2.39)	1.73 (1.17 to 02.55)	1.77 (0.97 to 02.78)	0.813

Data presented as number (proportion %), means ± standard deviations, or median (25th to 75th percentile) with *p* values calculated using chi‐squared, one‐way ANOVA, or Kruskal‐Wallis test, respectively.

Abbreviations: ADL, Activities of Daily Living; Agi, agitation; Anx, anxiety; Apa, apathy; BMI, body mass index; CDR, Clinical Dementia Rating; CIRS‐G, Cumulative Illness Rating Scale‐Geriatric; CU, cognitively unimpaired; Del, delusion; Dep, depression; GDS, Geriatric Depression Scale; HbA1C, hemoglobin A1c; HDL‐c, high‐density lipoprotein cholesterol; IADL, Instrumental Activities of Daily Living; Irr, irritability; LDL‐c, low density lipoprotein cholesterol; MCI, mild cognitive impairment; MMSE, Mini‐Mental State Examination; NPI, Neuropsychiatric Inventory; NPS, neuropsychiatric symptom; SCD, subjective cognitive decline; TSH, thyroid‐stimulating hormone.

Missing data: 429 for marital status; 235 for BMI; 113 for GDS; 241 for polypharmacy; 140 for CIRS‐G; 140 for CIRS‐G Comorbidity Index; 140 for CIRS‐G Severity Index; 241 for number of medications; 136 for stroke; 136 for myocardial infarction; 136 for cancer; 279 for thyroid functioning; 81 for cholesterol; 199 LDL‐c; 103 for HDL‐c; 87 for triglycerides; 85 for glucose; 997 for HbA1c; 962 for vitamin D; 95 for vitamin B12; 93 for TSH.

* *p* values are reported after false discovery rate adjustment for multiple comparisons.

### 3.1 NPS clusters by dementia status

To evaluate differences in NPS patterns across the dementia continuum, clustering was performed separately for participants with and without dementia (*n* = 632 and *n* = 602), revealing three clusters per group (Figure 1). In both, Cluster 0 (no/minimal NPSs) was the largest (dementia: *n* = 343,54.3%; dementia‐free: *n* = 351,58.3%). Cluster‐level symptom profiles are presented in Figure 2B,C, showing mean severity across all 12 NPI domains for each cluster in the dementia and dementia‐free[Table alz71255-tbl-0001] subgroups.

#### 3.1.1 Dementia subgroup: cluster characteristics

Cluster 0 (no/minimal NPSs) included 239 (69.7%) participants with mild dementia and 155 (45.2%) with clinical AD. Cluster 1 (depression‐anxiety‐apathy; *n* = 171, 27.1%) and Cluster 2 (delusions‐agitation‐depression‐anxiety‐irritability; *n* = 118, 18.7%) were predominant among participants with moderate to severe mixed dementia, with VaD more frequent in Cluster 1 (7%) than Cluster 2 (3.4%). Cluster 1 was mostly female (*n* = 131, 76.6%), with low education (85.4%), and had the highest medication use. Cluster 2 predominantly included older individuals, the highest underweight prevalence, low GDS‐based depressive symptoms, lower MMSE and IADL scores, highest NPI score, and lowest HDL‐c (Table 2).

**TABLE 2:** Demographic and clinical characteristics by NPS clusters in participants with dementia (*n* = 632).

Characteristics	Cluster 0 (no NPSs) *n* = 343	Cluster 1 (Dep‐Anx‐Apa) *n* = 171	Cluster 2 (Del‐Agi‐Dep‐Anx‐Irr) *n* = 118	*p* value^*^
**Demographic**				
Age years	81 (77 to 85)	81 (77 to 85)	83 (79 to 86)	0.082
Female sex	237 (69.1)	131 (76.6)	63 (53.4)	<0.001
Education				
Low (≤8 years)	266 (77.6)	146 (85.4)	91 (77.1)	0.209
High (>8 years)	77 (22.4)	25 (14.6)	27 (22.9)	
Marital status				
Unmarried	109 (48.0)	56 (50.9)	35 (49.3)	0.973
Married or in a relationship	118 (52.0)	54 (49.1)	36 (50.7)	
BMI	26.05 ± 4.30	26.33 ± 4.82	26.18 ± 5.33	0.968
Underweight (<18.5)	6 (1.7)	2 (1.2)	8 (6.8)	
Normal (≥18.5 to 25)	112 (32.7)	60 (35.1)	29 (24.6)	
Overweight (≥25 to 30)	100 (29.2)	51 (29.8)	36 (30.5)	0.099
Obesity (≥30)	54 (15.7)	29 (17.0)	16 (13.6)	
ADL score	5 (4 to 6)	4 (3 to 5)	4 (3 to 5)	0.133
IADL score	2 (1 to 4)	2 (1 to 4)	1 (1 to 3)	<0.001
Neuropsychiatric Inventory (NPI) total score	12 (6 to 16)	26 (21 to 36)	35.5 (29 to 45)	<0.001
**Cognitive status**				
MMSE score	18.08 ± 5.06	18.06 ± 5.01	16.64 ± 5.67	0.082
Dementia type				
Alzheimer's dementia	155 (45.2)	53 (31.0)	41 (34.7)	
Vascular dementia	24 (7.0)	17 (9.9)	4 (3.4)	0.022
Mixed dementia	136 (39.7)	83 (48.5)	63 (53.4)	
Cognitive impairment severity				
CDR = 1 (mild dementia)	239 (69.7)	112 (65.5)	64 (54.2)	
CDR = 2 (moderate dementia)	90 (26.2)	53 (31.0)	39 (33.1)	<0.001
CDR = 3 (severe dementia)	14 (4.1)	6 (3.5)	15 (12.7)	
**Medical conditions**				
Cumulative illness rating scale‐geriatric (CIRS‐G)				
Total score	9 (6 to 12)	11 (7.5 to 14)	10 (7 to 14)	0.019
Comorbidity index	1 (0 to 2)	1 (0 to 2)	1 (0 to 2)	0.082
Severity index	1.83 (1.5 to 2.0)	2 (1.6 to 2.1)	2 (1.6 to 2.2)	0.142
Number of medications	5 (3 to 7)	6 (4 to 8)	6 (4 to 8)	0.042
Polypharmacy (>5 drugs)	170 (57.4)	111 (72.5)	72 (67.3)	0.024
Stroke	17 (3)	6 (1)	6 (1)	0.934
Myocardial infarction	21 (3.7)	5 (0.9)	3 (0.5)	0.288
Diabetes	87 (13.8)	40 (6.3)	33 (5.2)	0.934
Cancer	46 (8)	27 (4.7)	16 (2.8)	0.956
Hyperlipidemia	238 (37.7)	122 (19.3)	80 (12.7)	0.956
Hypertension	256 (40.5)	133 (21)	91 (14.4)	0.934
Thyroid functioning				
Thyroid_normal	197 (57.4)	113 (66.1)	77 (65.3)	
Hypothyroidism	51 (14.9)	26 (15.2)	21 (17.8)	0.292
Hyperthyroidism	32 (9.3)	8 (4.7)	5 (4.2)	
**Blood biomarkers**				
Total cholesterol (mg/dL)	196 (166 to 230)	191 (162.5 to 230)	189 (157.2 to 227.7)	0.720
High cholesterol	141 (44.8)	72 (44.4)	43 (39.8)	0.934
HDL‐c (mg/dL)	56 (47 to 66.75)	56.5 (45 to 68)	49.5 (43 to 59.25)	0.019
Low HDL	36 (11.7)	27 (17.1)	19 (17.9)	0.2920.973
LDL‐c (mg/dL)	114 (89.7 to 138)	113.8 (86 to 144)	112.5 (87.2 to 141.2)	0.915
High LDL	29 (10.6)	19 (12.9)	14 (14.3)	
Triglycerides (mg/dL)	112 (81 to 141)	107 (85 to 143)	106 (81 to 138)	0.973
High triglycerides	65 (20.8)	35 (21.6)	23 (21.5)	0.973
Glucose (mg/dL)	99 (90 to 113)	98 (90 to 116)	99 (89 to 118)	0.973
HbA1C %	6.1 (5.7 to 7)	6.4 (5.8 to 7.5)	6.5 (5.7 to 7.7)	0.956
Poorly controlled (≥7.5)	11 (15.5)	9 (26.5)	7 (31.8)	0.342
Vitamin D (ng/mL)	21.35 (10.9 to 30.6)	23.7 (11 to 30.8)	16.5 (9.7 to 26.9)	0.501
Vitamin B12 (pg/mL)	241 (178.5 to 338)	252 (190 to 330.25)	218 (172 to 307.25)	0.582
TSH (µUI/mL)	1.72 (1.14 to 2.42)	1.67 (1.06 to 2.42)	1.7 (0.91 to 2.37)	0.934

Data presented as number (proportion %), means ± standard deviations, or median (25th–75th percentile) with p‐values calculated using Chi‐square, one‐way ANOVA, or Kruskal‐Wallis test, respectively.

Abbreviations: ADL, Activities of Daily Living; Agi, agitation; Anx, anxiety; Apa, apathy; BMI, body mass index; CDR, clinical dementia rating; CIRS‐G, cumulative illness rating scale‐geriatric; Del, delusion; Dep, depression; HbA1C, hemoglobin A1c; HDL‐c, high density lipoprotein; IADL, Instrumental Activities of Daily Living; Irr, irritability; LDL‐c, low density lipoprotein; MCI, mild cognitive impairment; MMSE, mini mental state examination; NPI, neuropsychiatric inventory; NPS, neuropsychiatric symptoms; SCD, subjective cognitive decline; TSH, thyroid‐stimulating hormone.

Missing data: 224 for marital status; 129 for BMI; 165 for polypharmacy; 69 for CIRS‐G; 69 for CIRS‐G Comorbidity Index; 69 for CIRS‐G Severity Index; 76 for number of medications; 60 for stroke; 60 for myocardial infarction; 60 for cancer; 102 for thyroid; 47 for cholesterol; 114 LDL‐c; 60 for HDL‐c; 50 for triglycerides; 44 for glucose; 505 for HbA1c; 488 for vitamin D; 55 for vitamin B12; 51 for TSH.

* *p* values are reported after FDR adjustment for multiple comparisons.

#### 3.1.2 Dementia‐free subgroup: cluster characteristics

Compared to Cluster 0 (no/minimal NPSs; *n* = 351, 58.27%), Cluster 1 (depression‐anxiety‐apathy‐irritability; *n* = 83, 13.8%) was characterized by the highest proportion of unmarried status, comorbidity burden (CIRS‐G median = 10 [IQR: 7 to 13]), medication use, and MCI (CDR 0.5). Cluster 2 (depression‐anxiety; *n* = 168, 27.9%) was predominantly female (77% vs 51% of Cluster 0) and had low education. Both Clusters 1 and 2 showed reduced ADL and/or IADL compared to Cluster 0, though still within unimpaired ranges (Table 3).

**TABLE 3:** Demographic and clinical characteristics by NPS clusters in dementia‐free participants (*n* = 602).

Characteristics	Cluster 0 (no NPS) *n* = 351	Cluster 1 (Dep‐Anx‐Apa‐ Irr) *n* = 83	Cluster 2 (Dep‐Anx) *n* = 168	*p* value^*^
**Demographic**				
Age years	78 (74 to 83)	80 (77 to 82)	78 (75.2 to 81)	0.237
Female sex	180 (51.3)	52 (62.7)	130 (77.4)	<0.001
Education				
Low (≤8 years)	215 (61.3)	53 (63.9)	123 (73.2)	0.117
High (>8 years)	136 (38.7)	30 (36.1)	45 (26.8)	
Marital status				
Unmarried	79 (35.4)	30 (53.6)	57 (48.3)	0.067
Married or in a relationship	144 (64.6)	26 (46.4)	61 (51.7)	
BMI	26.94 ± 4.28	26.89 ± 4.37	26.67 ± 4.46	0.988
Underweight (<18.5)	5 (1.7)	2 (2.9)	2 (1.5)	0.958
Normal (≥18.5 to 25)	92 (31.5)	22 (32.4)	46 (33.8)	
Overweight (≥25 to 30)	130 (44.5)	30 (44.1)	53 (39.0)	
Obesity (≥30)	65 (22.3)	14 (20.6)	35 (25.7)	
ADL score	6 (5 to 6)	5 (5 to 6)	5 (5 to 6)	<0.001
IADL score	6 (5 to 6)	4 (3 to 6)	6 (4 to 8)	<0.001
Depressive symptoms (GDS‐15 score ≥5)	96 (28.5)	52 (63.4)	120 (72.7)	<0.001
Neuropsychiatric inventory (NPI) total score	8 (4 to 13)	33 (27 to 42)	20 (16 to 26)	<0.001
**Cognitive status**				
MMSE score	26.06 ± 3.11	25.46 ± 3.17	25.89 ± 2.97	0.542
Clinical diagnosis				
Unimpaired	41 (11.7)	6 (7.2)	19 (11.3)	
SCD	53 (15.1)	4 (4.8)	22 (13.1)	0.237
MCI	257 (73.2)	73 (88.0)	127 (75.6)	
Cognitive severity				
CDR = 0 (CU and SCD)	94 (26.8)	10 (12.0)	41 (24.4)	0.088
CDR = 0.5 (MCI)	257 (73.2)	73 (88.0)	127 (75.6)	
**Medical conditions**				
CIRS‐G				
Total score	8 (5 to 11)	10 (7 to 13)	9 (6 to 12)	0.067
Comorbidity index	1 (0 to 1)	1 (0 to 2)	1 (0 to 1)	0.678
Severity index	1.67 (1.43 to 2)	1.8 (1.5 to 2)	1.71 (1.4 to 2)	0.542
Number of medications	5 (3 to 7)	6 (4 to 8)	5 (3 to 7)	0.410
Polypharmacy (>5 drugs)	130 (52.2)	41 (64.1)	75 (60.5)	0.378
Stroke	6 (2.0)	5 (6.6)	7 (3.4)	0.918
Myocardial infarction	11 (3.6)	1 (1.3)	5 (3.4)	0.836
Diabetes	91 (25.9)	23 (27.7)	34 (20.2)	0.542
Cancer	47 (15.5)	15 (19.7)	27 (18.5)	0.836
Hyperlipidemia	221 (63.0)	53 (63.9)	111 (66.1)	0.918
Hypertension	238 (67.8)	61 (73.5)	115 (68.5)	0.836
Thyroid functioning				
Thyroid_normal	180 (74.4)	40 (64.5)	88 (72.7)	
Hypothyroidism	42 (17.4)	14 (22.6)	19 (15.7)	0.782
Hyperthyroidism	20 (8.3)	8 (12.9)	14 (11.6)	
**Blood biomarkers**				
Total cholesterol (mg/dL)	191 (168.75 to 225.25)	190.5 (171.50 to 224.25)	196.5 (173.25 to 223.75)	0.839
High cholesterol	139 (42.1)	33 (42.3)	73 (45.6)	0.918
HDL‐c (mg/dL)	54 (46 to 65)	55.5 (47 to 67)	57 (49 to 68)	0.622
Low HDL	26 (8.0)	9 (11.5)	20 (12.7)	0.525
LDL‐c (mg/dL)	112 (93 to 142)	116 (95.5 to 140)	117 (96 to 140)	0.542
High LDL	40 (13.5)	9 (12.3)	20 (13.6)	0.987
Triglycerides (mg/dL)	102 (79.25 to 138.75)	120 (86 to 151.25)	109 (83 to 149)	0.237
High triglycerides	63 (19.2)	59 (75.6)	37 (23.3)	0.710
Glucose (mg/dL)	100 (92 to 117)	104 (94.5 to 119)	99 (88 to 113.5)	0.478
HbA1C %	6.3 (5.7 to 6.9)	6.1 (5.6 to 7)	6.4 (5.8 to 7)	0.958
Poorly controlled (≥7.5)	10 (14.5)	4 (20.0)	3 (14.3)	0.918
Vitamin D (ng/mL)	19.5 (13 to 33.1)	21.1 (12.7 to 34.9)	20.9 (12.7 to 30.9)	0.913
Vitamin B12 (pg/mL)	233.5 (180.5 to 328)	220 (176.5 to 323.5)	235.5 (188.2 to 309)	0.918
TSH (µUI/mL)	1.62 (1.08 to 2.44)	1.57 (1.12 to 2.42)	1.95 (1.25 to 2.64)	0.524

Data presented as number (proportion %), means ± standard deviations, or median (25th to 75th percentile) with *p* values calculated using chi‐squared, one‐way ANOVA, or Kruskal‐Wallis test, respectively.

Abbreviations: ADL, Activities of Daily Living; Agi, agitation; Anx, anxiety; Apa, apathy; BMI, body mass index; CDR, Clinical Dementia Rating; CIRS‐G, Cumulative Illness Rating Scale‐Geriatric; CU, cognitively unimpaired; Dep, depression; HbA1C, hemoglobin A1c; HDL‐c, high‐density lipoprotein cholesterol; IADL, Instrumental Activities of Daily Living; Irr, irritability; LDL‐c, low‐density lipoprotein cholesterol; MCI, mild cognitive impairment; MMSE, Mini‐Mental State Examination; NPI, Neuropsychiatric Inventory; NPS, neuropsychiatric symptoms; SCD, subjective cognitive decline; TSH, thyroid‐stimulating hormone.

Missing data: 205 for marital status; 106 for BMI; 18 for GDS; 76 for polypharmacy; 71 for CIRS‐G; 71 for CIRS‐G Comorbidity Index; 71 for CIRS‐G Severity Index; 165 for number of medications; 76 for stroke; 76 for myocardial infarction; 76 for cancer; 177 for thyroid; 34 for cholesterol; 85 LDL‐c; 43 for HDL‐c; 37 for triglycerides; 41 for glucose; 492 for HbA1c; 474 for vitamin D; 40 for vitamin B12; 42 for TSH.

* *p* values are reported after FDR adjustment for multiple comparisons.

### 3.2 Psychosocial and clinical determinants of NPS clusters

**FIGURE 3 alz71255-fig-0003:**
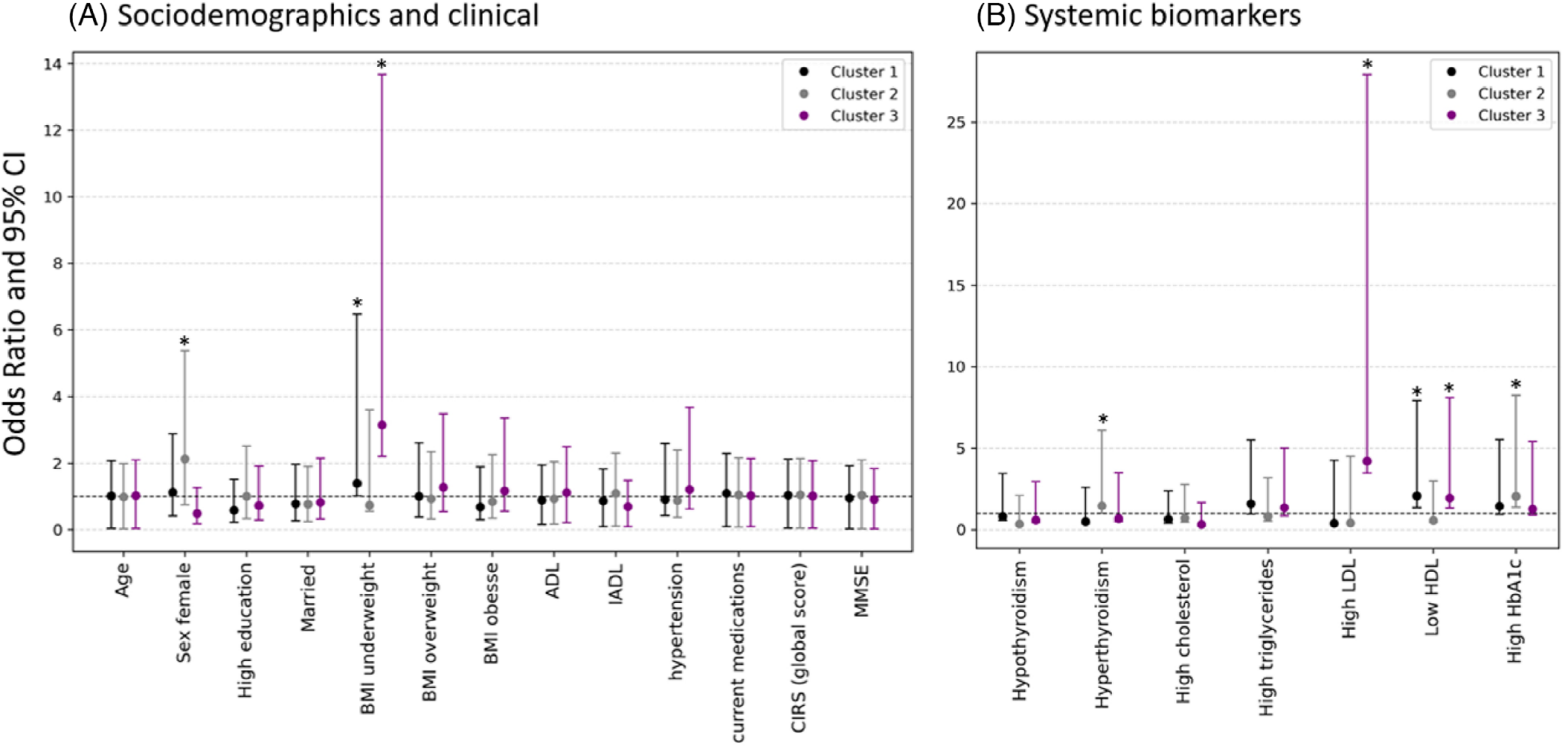
Sociodemographic, clinical, and systemic biomarker features of NPS clusters in the overall sample. The figure displays multinomial logistic regression models’ odds ratios and 95% confidence intervals (CIs) for associations between cluster allocation (outcome; with no/minimal NPS cluster as reference) and sociodemographic and clinical features (A) and systemic biomarkers (B) in overall sample. Cluster 1 depression‐anxiety‐apathy (circle black); Cluster 2 depression‐anxiety (gray circle); Cluster 3 delusions‐agitation‐irritability (circle purple) versus Cluster 0 no/minimal NPSs (reference). * Statistically significant odds ratios. ADL, Activities of Daily Living; BMI, body mass index; CIRS‐G, cumulative illness rating scale‐geriatric; HbA1C, hemoglobin A1c; HDL, high‐density lipoprotein; IADL, Instrumental Activities of Daily Living; LDL, low‐density lipoprotein; MMSE, Mini‐Mental State Examination.

**Overall sample** (*n* = 1234): Fully adjusted MLRMs show that, compared to Cluster 0, Cluster 1 was associated with lower HDL (OR = 2.07 [95% CI 1.34 to 7.93]) and underweight status (OR = 1.40 [95% CI 1.01 to 6.46]). Cluster 2 was associated with female sex (OR = 2.13 [95% CI 0.74 to 5.38]) and poor glycemic control (HbA1c ≥7.5%; OR = 2.04 [95% CI 1.37 to 8.25]). Cluster 3 showed increased odds of underweight (OR 3.14 [95% CI 2.20 to 13.7]), elevated LDL (OR 4.2 [95% CI 3.5 to 27.9]), and low HDL (OR 1.95 [95% CI 1.33 to 8.10]) (Figure 3; Table S4).

**FIGURE 4 alz71255-fig-0004:**
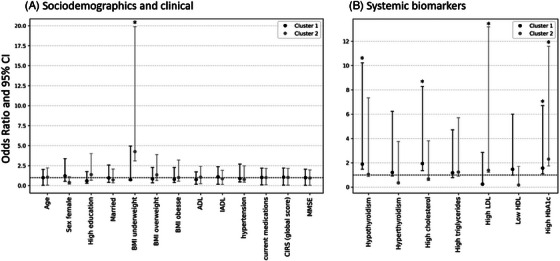
Sociodemographic, clinical, and systemic biomarker features of NPS clusters in the dementia group. The figure displays multinomial logistic regression models’ odds ratios and 95% confidence intervals (CIs) for associations between cluster allocation (outcome; with no/minimal NPS cluster as reference) and sociodemographic and clinical features (A) and systemic biomarkers (B) in dementia group. Cluster 1 depression‐anxiety‐apathy (black circle); Cluster 2 delusion‐agitation‐depression‐anxiety‐irritability (gray circle) versus Cluster 0 no/minimal NPS (reference). * Statistically significant odds ratios. ADL, Activities of Daily Living; BMI, body mass index; CIRS‐G, cumulative illness rating scale‐geriatric; HbA1C, hemoglobin A1c; HDL, high‐density lipoprotein; IADL, Instrumental Activities of Daily Living; LDL, low‐density lipoprotein; MMSE, Mini‐Mental State Examination.

**Dementia subgroup** (*n* = 632): Compared to Cluster 0, Cluster 1 was associated with higher odds of hypothyroidism (OR = 1.90 [95% CI 1.47 to 10.2]), high total cholesterol (OR = 1.95 [95% CI 1.35 to 8.29]), and greater HbA1c (OR = 1.56 [95% CI 1.09 to 6.70]). Cluster 2 showed a greater likelihood of being underweight (OR = 3.57 [95% CI 2.66 to 17.60]), poor glycemic control (HbA1c ≥7.5%; OR = 2.30 [95% CI 1.73 to 11.60]), and high LDL‐c (OR = 1.38 [95% CI 1.22 to 13.19]) (Figure 4; Table S5).

**FIGURE 5 alz71255-fig-0005:**
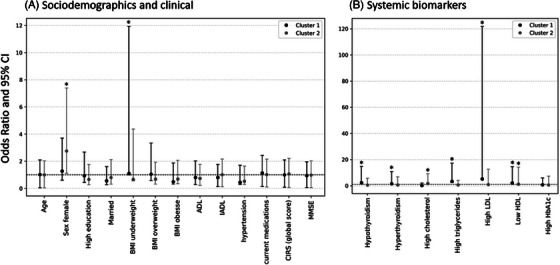
Sociodemographic, clinical, and systemic biomarker features of NPS clusters in the dementia‐free group. The figure displays multinomial logistic regression models’ odds ratios and 95% confidence intervals (CIs) for associations between cluster allocation (outcome; with no/minimal NPS cluster as reference) and sociodemographic and clinical features (A) and systemic biomarkers (B) in dementia‐free group. Cluster 1 depression‐anxiety‐apathy‐irritability (black circle); Cluster 2 depression‐anxiety (gray circle) versus Cluster 0 no/minimal NPS (reference). * Statistically significant odds ratios. ADL, Activities of Daily Living; BMI, body mass index; CIRS‐G, cumulative illness rating scale‐geriatric; HbA1C, hemoglobin A1c; HDL, high‐density lipoprotein; IADL, Instrumental Activities of Daily Living; LDL, low‐density lipoprotein; MMSE, Mini‐Mental State Examination.

**Dementia‐free subgroup** (*n* = 602): Relative to Cluster 0, Cluster 1 was strongly associated with elevated LDL (OR = 5.22 [95% CI 4.98 to 121.92]), high triglycerides (OR = 3.35 [95% CI 2.54 to 17.24]), thyroid dysfunction (hypothyroidism OR = 2.25 [95% CI 1.84 to 14.72], hyperthyroidism (OR = 1.46 [95% CI 1.23 to 10.78]), and low HDL (OR = 2.12 [95% CI 1.76 to 14.59]). Cluster 2 was linked to female sex (OR = 2.75 [95% CI 1.12 to 7.39]), high total cholesterol (OR = 1.66 [95% CI 1.30 to 9.24]), and low HDL (OR = 1.19 [95% CI 1.08 to 14.21]) (Figure 5; Table S6).

### 3.3 Sensitivity analysis

To assess the impact of anxiety and depression on NPS clustering, k‐means analysis was repeated without these symptoms.

Among 1234 participants, five clusters emerged: Cluster 0 (no/minimal NPSs; *n* = 561, 45.5%), Cluster 1 (agitation‐irritability; *n* = 137, 11.1%), Cluster 2 (delusion‐agitation‐irritability; *n* = 136, 11.0%), Cluster 3 (night‐time behavioral disturbances/sleep disorders; *n* = 119, 9.6%), and Cluster 4 (apathy; *n* = 281, 22.7%) (Figure 2D). Cluster 3 was mainly women of low education, in which 87% had dementia (Table S7). MLRMs showed no significant factors associated with Clusters 1, 3, and 4 versus Cluster 0; Cluster 2 was associated with underweight status (OR = 1.64 [95% CI 1.25 to 8.46]) (Table S8, Figure S3).

Dementia subgroup: Four clusters were identified. Compared to Cluster 0 (no/minimal NPSs), Cluster 1 (apathy) was associated with underweight (OR = 2.89 [95% CI 2.32 to 17.55]) and hyperthyroidism (OR = 2.52 [95% CI 1.49 to 8.69]); Cluster 2 (delusion) with higher education (OR = 2.08 [95% CI 1.12 to 6.60]) and high LDL (OR = 2.60 [95% CI 1.64 to 9.66]); Cluster 3 (agitation‐apathy‐irritability‐night‐time behavioral disturbances) with female sex (OR = 1.88 [95% CI 1.08 to 6.27]), BMI alterations (underweight OR = 1.76 [95% CI 1.50 to 13.79], overweight OR = 1.93 [95% CI 1.03 to 6.09], and obesity (OR = 1.92 [95% CI 1.11 to 6.50]) (Table S9, Figure S4).

Dementia‐free subgroup: Three clusters were identified. Compared to Cluster 0 (no/minimal NPSs), Cluster 1 (apathy) was associated with hypertension (OR = 1.86 [95% CI 1.04 to 6.06]). Cluster 2 (agitation‐irritability) was linked to obesity (OR = 2.12 [95% CI 1.36 to 8.04]) and low HDL (OR = 3.07 [95% CI 1.93 to 11.30]) (Table S10, Figure S5).

## 4 DISCUSSION

This cross‐sectional, memory clinic‐based study identified distinct NPS clusters across the cognitive spectrum, from unimpaired to dementia. Four main clusters emerged in the overall sample: (1) no/minimal NPSs, (2) depression‐apathy‐anxiety, (3) depression‐anxiety, and (4) delusions‐agitation‐irritability. Clusters differed in sex distribution, cognitive, functional, comorbidities, and metabolic profiles. Stratified analyses by dementia status showed comparable NPS patterns, but participants with dementia had more severe NPSs, greater functional impairment, and pronounced metabolic dysregulation. Notably, NPSs were common in the preclinical stage (42%). Clinical and metabolic factors – lipid abnormalities, glycemic control, thyroid dysfunction, and underweight – were differentially associated with NPS clusters. This suggests that late‐life NPSs may reflect a complex interplay of lifestyle, comorbidities, and metabolic factors, offering novel insights into prevention and management.

Half of our memory clinic participants exhibited clinically relevant NPSs, supporting prior work that NPS frequently accompany dementia and may precede it.^38^ This is consistent with the mild behavioral impairment, a neurobehavioral syndrome characterized by new‐onset, persistent behavioral change.^38^, ^39^ Our finding aligns with longitudinal studies indicating that depression, anxiety, and apathy can serve as early markers of neurodegeneration or cerebrovascular disease.^13^, ^40^, ^41^ The emergence of these symptoms in the preclinical phase may help predict cognitive progression to overt dementia – a hypothesis that warrants further investigation in population‐based, prospective cohorts. Recognizing and monitoring NPS early may help identify individuals at higher risk for cognitive decline, providing opportunities for timely interventions in acute and chronic conditions.

Our clustering[Fig alz71255-fig-0003], [Fig alz71255-fig-0004], [Fig alz71255-fig-0005] revealed partly overlapping yet distinct NPS patterns across dementia‐free and dementia groups. In dementia, two equally prevalent clusters emerged: one characterized by depression, anxiety, and apathy (Cluster 1), another behaviorally complex profile with delusions, agitation, depression, anxiety, and irritability (Cluster 2). Cluster 2 was linked to underweight, poor glycemic control, higher LDL‐c, and functional dependency. Co‐occurring NPSs may reduce appetite (leading to malnutrition), self‐care, and functional activity, worsening metabolic health; in turn, dysregulated glucose and lipid metabolism may promote inflammation and neurotransmitter imbalance, intensifying NPSs.^42^, ^43^ In the dementia‐free group, two clusters also emerged: one with depression, anxiety, apathy, and irritability, another with depression and anxiety. Cluster 1 was linked to greater multimorbidity and medication load, suggesting a possible relationship between polypharmacy, poorer health status, and NPS expression. Cluster 2 was linked to metabolic dysregulation (e.g., elevated LDL, triglycerides) and SCD. Sensitivity analyses excluding depression and anxiety confirmed stability of core clusters – especially those defined by apathy, agitation, and irritability – both in the overall sample and dementia‐status subgroups. Together, these findings suggest an evolving NPS profile along the dementia continuum: Affective symptoms may appear earlier during the preclinical stage, whereas complex patterns emerge later, with greater cognitive severity. Understanding how NPSs progress along the dementia continuum could inform stage‐specific strategies. Early detection of affective symptoms may enable preventive care and closer monitoring, while the emergence of psychotic symptoms may prompt advanced management. Longitudinal studies are needed to test this hypothesis.

Our clusters align with previously described NPS subsyndromes – affective, psychotic, hyperactivity/impulse control – with similar profiles observed across prodromal and overt dementia.^44^, ^45^, ^46^ A cohort study of ∼12,000 cognitively unimpaired older adults further showed that NPSs – particularly psychotic – predict higher dementia risk in AD and non‐AD particpants, supporting their potential as an early marker of neurodegeneration.^17^ A recent study using network analyses highlight structured co‐occurrence and bridge symptoms (e.g., anxiety‐sleep disturbance; apathy‐functional decline) linking NPSs and domain‐specific cognitive function in dementia participants.^10^ Within this framework, our clusters support previously described affective, psychotic, and hyperactivity profiles, while apathy emerges as a distinct pattern. We extend prior work by moving from symptom‐level associations to patient‐level clustering to identify clinically meaningful subgroups and explore associated health‐related factors. This study is an initial step toward the long‐term goal of defining NPS subgroups that may inform tailored interventions. We specifically aimed to determine whether – and which – individuals share similar NPS patterns, describe these patterns, and examine their links to health‐related factors, suggesting somatic correlates that may modulate symptom expression across the dementia continuum.

Identifying partly overlapping but distinct NPS clusters supports the idea that NPSs are not random and may reflect different mechanisms. For example, in the overall sample, the depression‐apathy‐anxiety pattern was more prevalent among women, individuals with low education, and those with a higher comorbidity burden – consistent with late‐life affective syndrome or the depression‐executive dysfunction dyad linked to frontal lobe vulnerability and cerebral small vessel disease.^47^ By contrast, the delusion‐agitation‐irritability pattern was linked to older age, male sex, and mixed/vascular dementia, consistent with orbitofrontal and temporoparietal cortical involvement underlying behavioral dysregulation in advanced cognitive disorders.^7^, ^48^ Neurodegenerative (e.g., AD) and cerebrovascular (e.g., small vessel disease) pathology may contribute differently to NPSs before dementia onset – e.g., AD often linked to depression/anxiety, vascular pathology to apathy.^13^, ^41^ We could not directly test mechanisms due to absent biomarkers and neuroimaging. Future work incorporating MRI and comprehensive neuropsychological measures will inform about mechanistic pathways more robustly.

NPS clusters appeared to be influenced by a multifactorial interplay of psychosocial and health‐related factors. Female sex, low education, underweight, dyslipidemia, and poor glycemic control were key factors for specific NPS clusters. Female sex and poor glycemic control were particularly associated with anxiety‐depression clusters, consistent with greater vulnerability to depression in women, with mild anxiety as a potential preclinical symptom.^11^, ^49^ The association between low education and NPSs may reflect reduced cognitive reserve and coping strategies.^50^ We hypothesize that lower education reduces the threshold for pathology to manifest behaviorally, enabling prodromal NPSs such as depression and anxiety to emerge earlier, even before cognitive deficits become clinically evident – a hypothesis to be tested. Dyslipidemia emerged as a cross‐cutting feature across clusters: co‐elevated LDL and triglycerides were linked to psychosis‐like clusters in dementia. This may reflect impaired health management as cognition worsens or, conversely, early neuropathological changes that trigger behavioral alterations. Supporting this, we previously showed that altered glucose and lipid metabolism with systemic inflammation were associated with older‐appearing brains, especially in men,^51^ suggesting these biological processes may also shape NPS profiles during preclinical stages. Underweight and hypothyroidism were likewise associated with psychosis‐like clusters, reinforcing nutritional and endocrine vulnerabilities in neuropsychiatric phenotypes.^52^, ^53^ Finally, apathy emerged as a distinct cluster in both dementia and dementia‐free groups – distinctly associated with hyperthyroidism in dementia and hypertension in dementia‐free – supporting apathy as a primary NPS rather than secondary to mood disorders. Future research should clarify apathy's relationship to mood disorders – whether they stem from shared mechanisms, co‐exist independently, or influence each other – and their individual and joint impact on brain and cognition.

This study has notable strengths. While previous work identified NPS clusters using different methods, we are among the first to explicitly link NPS clusters to health‐related factors. Our large, clinically well‐characterized memory clinic cohort benefits from routinely multidimensional geriatric assessments integrated into clinical practice. Our cohort includes individuals with referrals for cognitive complaints due to broader age‐related neuropathologies, enhancing heterogeneity and ecological validity. Access to extensive health‐related data, including metabolic biomarkers, deepens interpretation of NPS patterns. Methodologically, k‐means provided a robust, data‐driven approach for identifying naturally occurring NPS profiles; the sample size enabled well‐powered regression analyses with adjustment for confounders.

Limitations include the cross‐sectional design, which precluded temporal inference about NPS and cognitive decline, and the inability to determine whether clinically unimpaired individuals with certain NPS patterns were at higher risk of progressing to dementia. We cannot fully distinguish pre‐existing depression/anxiety from prodromal manifestations, although our memory clinic's referral procedure reduces inclusion of chronic psychiatric disorders. Caregiver‐administered NPI interviews may introduce reporting bias and misclassification, potentially underestimating observed associations. Nonetheless, integration of clinical diagnoses, functional assessments, and biomarker data likely mitigates this risk, enhancing overall validity. The absence of neuroimaging limits linkage of NPSs to neurodegeneration and cerebrovascular mechanisms. Alternative clustering (e.g., hierarchical clustering, DBSCAN) could offer complementary insights; however, our choice of k‐means prioritized interpretability and computational efficiency. The dementia‐free group included SCD and MCI; while reflecting real‐world memory clinic populations and enhancing external validity, findings could differ if restricted to cognitively unimpaired versus MCI individuals. The predominantly Caucasian sample and recruitment from specialized clinical settings may limit generalizability to more diverse, community‐based populations.

Identifying distinct NPS clusters and their associations with metabolic, nutritional, and sociodemographic factors offers clinically meaningful insights into NPS heterogeneity across the dementia continuum. By linking NPS clusters to modifiable factors, our findings support multidimensional assessments extending beyond cognition and behaviors. For example, affective NPSs alongside dyslipidemia or poor glycemic control may warrant closer monitoring and tailored interventions addressing both neuropsychiatric and somatic vulnerabilities, whereas psychosis‐like NPS profiles co‐occurring with underweight and functional dependency highlight the need for integrated care combining behavioral management, nutritional support, and frailty assessment. These implications are relevant for preventive strategies in pre‐dementia stages and comprehensive care planning in overt dementia. Future work needs to replicate NPS patterns in diverse populations and test whether targeting metabolic risk can mitigate NPSs, with consequent cognitive benefits. Incorporating NPS, metabolic, and lifestyle dimensions into patient stratification offers a personalized approach to refining dementia therapeutic and preventive strategies.

## CONFLICT OF INTEREST STATEMENT

The authors declare no conflicts of interest. Author disclosures are available in the Supporting Information.

## Supporting information



Supporting Information

Supporting Information
