# Supplementary material for: Neuropsychiatric symptoms in preclinical and clinically manifest dementia: clusters and their health determinants
Source: Alzheimers Dement. 2026 Mar 8;22(3):e71255. doi: 10.1002/alz.71255 (PMC12967499; doi:10.1002/alz.71255)
Supplement: Supplementary file 1 — Supporting Information [file ALZ-22-e71255-s002.docx]

**SUPPLEMENTARY MATERIAL**

| **Supplementary Table 1—**Demographic and clinical characteristics of the 1234 participants in the GERICO cohort | Page 2 |
| --- | --- |
| **Supplementary Table 2 —**Descriptive data on neuropsychiatric symptoms (NPI domains) by dementia status | Page 5 |
| **Supplementary Table 3—**Cluster centroids (mean NPI domain scores) across models (overall sample, dementia-free, dementia, and overall sample excluding depression/anxiety) | Page 6 |
| **Supplementary Table 4** —Associations between cluster allocation and potential sociodemographic and health-related determinants in overall sample | Page 7 |
| **Supplementary Table 5**—Associations between cluster allocation and potential sociodemographic and health-related determinants in dementia group. | Page 9 |
| **Supplementary Table 6**—Associations between cluster allocation and potential sociodemographic and health-related determinants in dementia-free group | Page 11 |
| **Supplementary Table 7—**Demographic and clinical characteristics by clusters of neuropsychiatric symptoms in the GERICO cohort (n=1234) excluded Depression and Anxiety Symptoms | Page 13 |
| **Supplementary Table 8—**Associations between cluster allocation and potential sociodemographic and health-related determinants in overall sample excluding anxiety and depression | Page 16 |
| **Supplementary Table 9—**Associations between cluster allocation and potential sociodemographic and health-related determinants in dementia group excluding anxiety and depression | Page 18 |
| **Supplementary Table 10—**Associations between cluster allocation and potential sociodemographic and health-related determinants in dementia free group excluding anxiety and depression | Page 20 |
| **Supplementary Figure 1—**Optimal number of clusters for the overall sample, dementia group, and dementia-free group. | Page 21 |
| **Supplementary Figure 2—**Agreement between Euclidean k-means and alternative distance-based clustering solutions. | Pag 23 |
| **Supplementary Figure 3—**Sociodemographic, clinical and systemic biomarkers feature of the NPS clusters in the overall sample excluding depression and anxiety | Page 24 |
| **Supplementary Figure 4—**Sociodemographic, clinical and systemic biomarkers feature of the NPS clusters in the dementia group excluding depression and anxiety | Page 25 |
| **Supplementary Figure 5—**Sociodemographic, clinical and systemic biomarkers feature of the NPS clusters in the dementia free group excluding depression and anxiety | Page 26 |

**Supplementary Table 1**—Demographic and clinical characteristics of the 1234 participants in the GERICO cohort

| **Characteristics** | **Total (n=1234)** | |
| --- | --- | --- |
| **Demographic** | | |
| Age, years | 80 (76–84) | |
| Female sex | 793 (64.3) | |
| Education. years | 5 (5-11) | |
| Low (≤ 8 years) | 894 (72.4) | |
| High (> 8 years) | 340 (27.6) | |
| Marital status | 805 (65.2) | |
| Unmarried | 366 (45.5) | |
| Married or in a relationship | 439 (54.5) | |
| BMI | 26.55 ± 4.5 | |
| Underweight (<18.5) | 25 (2.5) | |
| Normal (≥18.5–25) | 361 (36.1) | |
| Overweight (≥25–30) | 400 (40.0) | |
| Obesity (≥30) | 213 (21.3) | |
| ADL score | 5 (4-6) | |
| IADL score | 4 (2-6) | |
| Depressive symptoms (GDS-15 score ≥5) | 523 (46.7) | |
| **Cognitive status** | | |
| MMSE score | 21.77 ± 5.91 | |
| Unimpaired | 66 (5.3) | |
| SCD | 79 (6.4) | |
| MCI | 457 (37.0) | |
| Dementia diagnosis | 632 (51.2) | |
| CDR= 0 (UC and SCD) | 145 (11.8) | |
| CDR= 0.5 (MCI) | 457 (37.0) | |
| CDR= 1 (Mild dementia) | 415 (33.6) | |
| CDR= 2 (Moderate dementia) | 182 (14.7) | |
| CDR= 3 (Severe dementia) | 35 (2.8) | |
| **Neuropsychiatric symptoms** | | |
| Neuropsychiatric Inventory (NPI) |  | |
| Total score | 16.00 (8-26) | |
| Item-specific scores |  | |
| Hallucination | 151 (12.1) | 4.00 (2-6) |
| Delusion | 245 (19.6) | 4.00 (3-6) |
| Agitation/Aggression | 367 (29.4) | 4.00 (3-6) |
| Depression/Dysphoria | 829 (66.4) | 6.00 (4-9) |
| Anxiety | 775 (62.1) | 6.00 (4-8) |
| Apathy/Indifference | 559 (44.8) | 4.00 (4-8) |
| Elation/Euphoria | 51 (4.1) | 4.00 (2-6) |
| Irritability/Lability | 559 (44.8) | 4.00 (3-6) |
| Disinhibition | 141 (11.3) | 4.00 (2-4) |
| Motor Disturbance | 147 (11.8) | 4.00 (4-8) |
| Nighttime Behaviors | 385 (30.8) | 4.00 (2-6) |
| Appetite/Eating | 242 (19.4) | 4.00 (2-6) |
| **Medical conditions** | | |
| Cumulative Illness Rating Scale-Geriatric (CIRS_G)  Total Score | 9 (6-12) | |
| Comorbidity index | 1 (0-2) | |
| Severity Index | 1.8 (1.5-2) | |
| Number of medication | 5 (3-7) | |
| Polypharmacy (>5 drugs) | 599 (60.3) | |
| Stroke | 47 (3.8) | |
| Myocardial infarction | 46 (3.7) | |
| Diabetes | 308 (25.0) | |
| Cancer | 178 (14.4) | |
| Hyperlipidemia | 825 (66.9) | |
| Hypertension | 894 (72.4) | |
| Thyroid functioning |  | |
| Thyroid normal | 695 (56.3) | |
| Hypothyroidism | 173 (14.0) | |
| Hyperthyroidism | 87 (7.1) | |
| **Blood biomarkers** | | |
| Total Cholesterol (mg/dl) | 193 (167-227) | |
| High Cholesterol | 501 (43.5) | |
| LDL-c (mg/dl) | 113.8 (91-140) | |
| High LDL | 131 (12.7) | |
| HDL-c (mg/dl)  Low HDL | 55 (46-66)  137 (12.1) | |
| Triglycerides (mg/dl)  High triglycerides | 107 (82-142)  242 (21.1) | |
| Glucose (mg/dL) | 100 (90-115) | |
| HbA1C % | 6.3 (5.7-7.1) | |
| Poor controlled (≥7.5) | 44 (18.6) | |
| Vitamin D (ng/mL) | 20.55 (11.82-30.77) | |
| Vitamin B12 (pg/ml) | 236 (182-327) | |
| TSH (μUI/ml) | 1.70 (1.10-2.44) | |

Data presented as number (proportion. %), means ± standard deviations, or median (25th–75th percentile).

Abbreviations: BMI, body mass index; ADL, activities of daily living; IADL, instrumental activities of daily living; GDS, geriatric depression scale; MMSE, mini mental state examination; SCD, subjective cognitive decline; MCI, mild cognitive impairment; CDR, clinical dementia rating; NPI, neuropsychiatric inventory; CIRS_G, cumulative illness rating scale-geriatric; LDL, low density lipoprotein; HDL, high density lipoprotein; HbA1C, hemoglobin A1c; TSH, thyroid-stimulating hormone.

Missing data: 429 for Marital Status. 235 for BMI. 241 for polypharmacy. 113 for GDS. 140 for CIRS_G. 140 for CIRS_G_Comorbidity Index. 140 for CIRS_G_Severity Index. 241 for Number of medications. 136 for Stroke. 136 for Myocardial infarction. 136 for Cancer. 279 for Thyroid functioning. 81 for Cholesterol. 199 LDL-c. 103 for HDL-c. 87 for Triglycerides. 85 for Glucose. 997 for HbA1c. 962 for Vitamin D. 95 for Vitamin B12. 93 for TSH.

**Supplementary Table 2—**Descriptive data on neuropsychiatric symptoms (NPI domains) by dementia status.

| **NPI domains** | **Dementia free group**  **(n=602)** | | **Dementia group**  **(n=632)** | |
| --- | --- | --- | --- | --- |
| **Total score. median (IQR)** | 14.00 (7-24) | | 18 (10-29.75) | |
|  |  | |  | |
| **Item-specific scores** | **N (%)** | **Median (IQR)** | **N (%)** | **Median (IQR)** |
| Delusion | 57 (9.5) | 4.00 (2-6) | 186 (29.4) | 4.00 (4-6) |
| Hallucination | 24 (4.0) | 4.00 (2-5.5) | 125 (19.8) | 4.00 (2-6) |
| Agitation/Aggression | 131 (21.8) | 4.00 (4-6) | 232 (36.7) | 4.00 (3-6) |
| Depression/Dysphoria | 415 (68.9) | 6.00 (4-9) | 410 (64.9) | 6.00 (4-9) |
| Anxiety | 400 (66.4) | 6.00 (4-9) | 368 (58.2) | 6.00 (4-6) |
| Apathy/Indifference | 235 (39.0) | 4.00 (3-6) | 316 (50) | 6.00 (4-8) |
| Elation/Euphoria | 17 (2.8) | 4.00 (2-6) | 33 (5.2) | 3.00 (2-4) |
| Irritability/Lability | 237 (39.4) | 4.00 (3-6) | 314 (49.7) | 4.00 (3-6) |
| Disinhibition | 45 (7.5) | 4.00 (2-4) | 90 (14.2) | 4.00 (2-6) |
| Motor Disturbance | 28 (4.7) | 4.00 (2-6) | 119 (18.8) | 4.00 (4-9) |
| Nighttime Behaviors | 178 (29.6) | 4.00 (2-6) | 203 (32.1) | 4.00 (3-8) |
| Appetite/Eating | 91 (15.1) | 4.00 (2-8) | 147(23.3) | 4.00 (2-6) |

For each group, n (%) indicates the number of participants with the presence of the specific neuropsychiatric symptom. Median (IQR) scores are reported for each NPI domain when present (range: 0–12).

Abbreviations: IQR, interquartile range; NPI, Neuropsychiatric Inventory

**Supplementary Table 3—**Cluster centroids (mean NPI domain scores) across models (overall sample, dementia-free, dementia, and overall sample excluding depression/anxiety).

| **Cluster** | **Del** | **Hall** | **Agi** | **Dep** | **Anx​** | **Eup** | **Apa** | **Dis** | **Irr​** | **MotDist** | **NightB** | **EatDis** |
| --- | --- | --- | --- | --- | --- | --- | --- | --- | --- | --- | --- | --- |
| **Overall sample** | | | | | | | | | | | | |
| C0 ​ | -0.35​ | -0.58​ | -0.23​ | 1.08​ | 1.14​ | -0.81​ | 0.43​ | -0.65​ | 0.53​ | -0.58​ | -0.08​ | -0.63​ |
| C1​ | -0.10​ | -0.19​ | 0.10​ | **7.35​** | **3.78​** | -0.91​ | **7.71**​ | -0.73​ | 1.13​ | -0.23​ | 1.14​ | 1.48​ |
| C2​ | -0.47​ | -0.79​ | 0.28​ | **6.88​** | **7.10​** | -0.86​ | -0.09​ | -0.67​ | 1.54​ | -0.55​ | 1.65​ | -0.17​ |
| C3​ | **3.46**​ | 1.28​ | **5.85**​ | 2.82​ | 2.75​ | -0.57​ | 2.36​ | 0.96​ | **5.85​** | 1.78​ | 2.63​ | 1.05​ |
| **Dementia-free group** | | | | | | | | | | | | |
| C0​ | -0.62​ | -0.79​ | -0.11​ | 1.34​ | 0.74​ | -0.81​ | 0.56​ | -0.57​ | 1.06​ | -0.81​ | 0.03​ | -0.58​ |
| C1​ | -0.32​ | -0.68​ | 1.73​ | **8.03​** | **5.01​** | -0.79​ | **6.61​** | -0.47​ | **3.24​** | -0.37​ | 2.95​ | 2.44​ |
| C2​ | -0.47​ | -0.86​ | 0.08​ | **6.06​** | **7.06​** | -0.95​ | 0.04​ | -0.76​ | 0.61​ | -0.77​ | 0.65​ | -0.63 |
| **Dementia group** | | | | | | | | | | | | |
| C0​ | -0.04​ | -0.26​ | 0.09​ | 1.37​ | 1.59​ | -0.75​ | 0.67​ | -0.55​ | 0.75​ | -0.18​ | 0.34​ | -0.50​ |
| C1​ | -0.05​ | -0.18​ | -0.02​ | **7.61​** | **4.92​** | -0.91​ | **5.83**​ | -0.68​ | 1.36​ | -0.11​ | 1.64​ | 1.14​ |
| C2​ | **4.39**​ | 1.73​ | **5.92​** | **3.33​** | **3.28​** | -0.59​ | 1.87​ | 0.87​ | **5.37​** | 2.18​ | 2.76​ | 0.92 |
| **Overall sample excluding depression and anxiety** | | | | | | | | | | | | |
| C0​ | -0.75​ | -0.82​ | -0.42​ |  |  | -0.80​ | -0.63​ | -0.73​ | 0.32​ | -0.75​ | -0.34​ | -0.68​ |
| C1​ | -0.12​ | -0.51​ | **6.09​** |  |  | -0.59​ | 2.67​ | 0.57​ | **6.89**​ | 0.44​ | 0.95​ | 0.80​ |
| C2​ | **6.55​** | 2.24​ | **3.36**​ |  |  | -0.60​ | 0.95​ | 0.56​ | **3.08**​ | 1.57​ | 1.34​ | 0.04​ |
| C3​ | -0.38​ | 0.06​ | 0.27​ |  |  | -0.92​ | 2.03​ | -0.80​ | 1.78​ | -0.02​ | **7.49​** | 1.52​ |
| C4​ | -0.39​ | -0.52​ | -0.31 |  |  | -0.95​ | **6.72​** | -0.65​ | 0.79​ | -0.29​ | -0.34​ | 0.47 |

Values are cluster centroids, i.e., the mean score for each NPI domain among individuals assigned to that cluster. These centroids reflect the multidimensional symptom profiles that define each cluster and allow for comparison of the relative prominence of individual neuropsychiatric symptoms (NPS) across clusters (C). Values selected for inclusion in the cluster are shown in bold.

Abbreviations: Del, delusion; Hall, hallucination; Agi, agitation; Dep, depression; Anx, anxiety; Eup, euphoria; Apa, apathy; Dis, disinhibition; Irr, irritability; MotDist, motor disturbance; NightB, nighttime behavioral disturbances; EatDis, appetite/eating disorder.

**Supplementary Table 4** —Associations between cluster allocation and potential sociodemographic and health-related determinants in overall sample.

|  | **Cluster 1** (**n=279)** | | **Cluster 2 (n=224)** | | **Cluster 3 (n=174)** | |
| --- | --- | --- | --- | --- | --- | --- |
|  | **(Dep-Anx-Apa)** | | **(Dep-Anx)** | | **(Del-Agi-Irr)** | |
|  | **OR (95%CI)** | **p-values** | **OR (95%CI)** | **p-values** | **OR (95%CI)** | **p-values** |
| Age. year | 1.02 (0.03-2.07) | 0.007 | 0.98 (0.03-1.98) | 0.463 | 1.03 (0.04-2.09) | 0.004 |
| Female sex | 1.12 (0.4-2.87) | 0.051 | 2.13 (0.74-5.38) | <0.001 | 0.48 (0.18-1.26) | 0.001 |
| Education High (>8 years) | 0.58 (0.22-1.51) | 0.007 | 1.01 (0.33-2.51) | 0.106 | 0.72 (0.28-1.91) | 0.061 |
| Marital Status. Married or in a relationship | 0.78 (0.27-1.96) | 0.040 | 0.77 (0.25-1.89) | 0.021 | 0.82 (0.31-2.15) | 0.826 |
| BMI underweight | 1.4 (1.01-6.46) | 0.810 | 0.73 (0.54-3.6) | 0.168 | 3.14 (2.2-13.67) | 0.782 |
| BMI overweight | 1 (0.37-2.6) |  | 0.92 (0.32-2.34) |  | 1.28 (0.54-3.48) |  |
| BMI obese | 0.68 (0.3-1.89) |  | 0.84 (0.34-2.25) |  | 1.16 (0.54-3.35) |  |
| ADL score | 0.88 (0.15-1.94) | <0.001 | 0.92 (0.16-2.05) | 0.769 | 1.11 (0.21-2.48) | <0.001 |
| IADL score | 0.86 (0.09-1.82) | <0.001 | 1.09 (0.1-2.29) | 0.076 | 0.69 (0.09-1.47) | <0.001 |
| Hypertension | 0.9 (0.42-2.58) | 0.129 | 0.88 (0.37-2.38) | 0.411 | 1.21 (0.62-3.67) | 0.137 |
| Number of medications | 1.09 (0.09-2.28) | <0.001 | 1.04 (0.08-2.16) | 0.021 | 1.02 (0.09-2.14) | 0.091 |
| CIRS_G (total score) | 1.04 (0.05-2.12) | <0.001 | 1.05 (0.05-2.14) | 0.004 | 1.01 (0.05-2.07) | 0.006 |
| MMSE score | 0.95 (0.02-1.92) | <0.001 | 1.03 (0.03-2.09) | 0.034 | 0.91 (0.03-1.84) | <0.001 |
| Hypothyroidism | 0.83 (0.57-3.46) | 0.706 | 0.35 (0.28-2.09) | 0.743 | 0.59 (0.44-2.95) | 0.912 |
| Hyperthyroidism | 0.51 (0.39-2.61) |  | 1.46 (1-6.08) |  | 0.68 (0.52-3.5) |  |
| High Cholesterol | 0.64 (0.41-2.39) | 0.287 | 0.72 (0.47-2.77) | 0.704 | 0.34 (0.25-1.65) | 0.047 |
| High triglycerides | 1.6 (0.94-5.51) | 0.229 | 0.83 (0.54-3.2) | 0.326 | 1.34 (0.85-5) | 0.219 |
| High LDL | 0.41 (0.36-4.26) | 0.503 | 0.42 (0.37-4.51) | 0.570 | 4.22 (3.47-27.91) | 0.968 |
| Low HDL | 2.07 (1.34-7.93) | 0.017 | 0.58 (0.44-3) | 0.115 | 1.95 (1.33-8.1) | 0.010 |
| Poor controlled HbA1c (≥7.5) | 1.43 (0.93-5.51) | 0.392 | 2.04 (1.37-8.25) | 0.135 | 1.25 (0.87-5.41) | 0.274 |

Estimated Odds Ratios (OR) and 95% Confidence Intervals (95% CI) for associations between cluster allocation (outcome; with the no/minimal NPS cluster as the reference) and potential sociodemographic and health-related determinants in overall sample. Reference group for the health-related factors: Male sex; Education Low (≤ 8 years); Unmarried for marital status; BMI normal for BMI categories; Absence of hypertension; Thyroid normal for thyroid functioning categories; HbA1c <7.5 for HbA1c %.

Abbreviations: NPS, neuropsychiatric symptoms; Del, delusion; Agi, agitation; Dep, depression; Anx, anxiety; Apa, apathy; Irr, irritability; BMI, body mass index; ADL, activities of daily living; IADL, instrumental activities of daily living; CIRS-G, cumulative illness rating scale-geriatric; MMSE, mini mental state examination; LDL, low density lipoprotein; HDL, high density lipoprotein; HbA1C, hemoglobin A1c.

**Supplementary Table 5**—Associations between cluster allocation and potential sociodemographic and health-related determinants in dementia group.

|  | **Cluster 1 (n = 171)** | | **Cluster 2 (n = 118)** | |
| --- | --- | --- | --- | --- |
|  | **(Dep-Anx-Apa)** | | **(Del-Agi-Dep-Anx-Irr)** | |
|  | **OR (95%CI)** | **p-values** | **OR (95%CI)** | **p-values** |
| Age. year | 0.99 (0.04-2.03) | 0.798 | 1.07 (0.05-2.19) | 0.002 |
| Female sex | 1.21 (0.53-3.36) | 0.075 | 0.38 (0.18-1.1) | 0.002 |
| Education. High (> 8 years) | 0.6 (0.29-1.74) | 0.036 | 1.38 (0.65-4.01) | 0.923 |
| Marital Status. Married or in a relationship | 0.97 (0.38-2.57) | 0.620 | 0.71 (0.33-2.05) | 0.851 |
| BMI underweight | 0.86 (0.7-5.68) | 0.024 | 3.57 (2.66-17.6) | 0.060 |
| BMI overweight | 1.04 (0.44-2.86) |  | 1.18 (0.57-3.46) |  |
| BMI obese | 0.83 (0.41-2.47) |  | 0.95 (0.52-3.05) |  |
| ADL score | 0.8 (0.16-1.8) | <0.001 | 1.02 (0.23-2.33) | 0.009 |
| IADL score | 1.07 (0.15-2.33) | 0.097 | 0.88 (0.15-1.95) | 0.001 |
| Hypertension | 0.74 (0.38-2.27) | 0.435 | 0.91 (0.52-3.02) | 0.591 |
| Number of medications | 1.02 (0.1-2.16) | 0.003 | 1 (0.11-2.12) | 0.126 |
| CIRS_G (total score) | 1.06 (0.05-2.17) | <0.001 | 1.04 (0.06-2.14) | 0.063 |
| MMSE score | 1 (0.04-2.04) | 0.966 | 0.95 (0.04-1.94) | 0.010 |
| Hypothyroidism | 1.9 (1.47-10.23) | 0.969 | 1.05 (0.88-7.35) | 0.774 |
| Hyperthyroidism | 1.21 (0.92-6.23) |  | 0.35 (0.31-3.75) |  |
| High Cholesterol | 1.95 (1.35-8.29) | 0.947 | 0.68 (0.53-3.8) | 0.372 |
| High triglycerides | 1.18 (0.78-4.71) | 0.832 | 1.24 (0.9-5.7) | 0.873 |
| High LDL | 0.24 (0.22-2.86) | 0.480 | 1.38 (1.22-13.19) | 0.333 |
| Low HDL | 1.46 (0.99-6) | 0.107 | 0.17 (0.15-1.7) | 0.103 |
| Poor controlled HbA1c (≥7.5) | 1.56 (1.09-6.7) | 0.183 | 2.3 (1.73-11.6) | 0.092 |

Estimated Odds Ratios (OR) and 95% Confidence Intervals (95% CI) for associations between cluster allocation (outcome; with the no/minimal NPS cluster as the reference) and potential sociodemographic and health-related determinants in dementia group. Reference group for the health-related factors: Male sex; Education Low (≤ 8 years); Unmarried for marital status; BMI normal for BMI categories; Absence of hypertension; Thyroid normal for thyroid functioning categories; HbA1c <7.5 for HbA1c %.

Abbreviations: NPS, neuropsychiatric symptoms; Del, delusion; Agi, agitation; Dep, depression; Anx, anxiety; Apa, apathy; Irr, irritability; BMI, body mass index; ADL, activities of daily living; IADL, instrumental activities of daily living; CIRS-G, cumulative illness rating scale-geriatric; MMSE, mini mental state examination; LDL, low density lipoprotein; HDL, high density lipoprotein; HbA1C, hemoglobin A1c.

**Supplementary Table 6**—Associations between cluster allocation and potential sociodemographic and health-related determinants in dementia-free group.

|  | **Cluster 1 (n = 83)** | | **Cluster 2 (n=168)** | |
| --- | --- | --- | --- | --- |
|  | **(Dep-Anx-Apa_Irr)** | | **(Dep-Anx)** | |
|  | **OR (95%CI)** | **p-values** | **OR (95%CI)** | **p-values** |
| Age. year | 1.02 (0.05-2.09) | 0.077 | 1 (0.03-2.03) | 0.973 |
| Female sex | 1.28 (0.6-3.7) | 0.062 | 2.75 (1.12-7.39) | <0.001 |
| Education. High (> 8 years) | 0.93 (0.43-2.67) | 0.662 | 0.66 (0.26-1.76) | 0.007 |
| Marital Status. Married or in a relationship | 0.55 (0.26-1.61) | 0.013 | 0.8 (0.31-2.12) | 0.021 |
| BMI underweight | 1.1 (0.99-11.93) | 0.084 | 0.66 (0.54-4.38) | 0.041 |
| BMI overweight | 1.04 (0.57-3.33) |  | 0.68 (0.31-1.92) |  |
| BMI obese | 0.49 (0.32-1.87) |  | 0.69 (0.35-2.07) |  |
| ADL score | 0.8 (0.27-2.02) | 0.002 | 0.74 (0.21-1.78) | 0.011 |
| IADL score | 0.8 (0.13-1.75) | <0.001 | 1.01 (0.12-2.16) | 0.913 |
| Hypertension | 0.44 (0.29-1.7) | 0.315 | 0.53 (0.28-1.65) | 0.883 |
| Number of medications | 1.13 (0.15-2.42) | 0.018 | 1.02 (0.1-2.15) | 0.150 |
| CIRS_G (total score) | 1 (0.08-2.09) | 0.010 | 1.07 (0.07-2.21) | 0.039 |
| MMSE score | 0.94 (0.07-1.96) | 0.117 | 0.98 (0.06-2.03) | 0.569 |
| Hypothyroidism | 2.25 (1.84-14.72) | 0.981 | 0.53 (0.48-5.7) | 0.709 |
| Hyperthyroidism | 1.46 (1.23-10.78) |  | 0.65 (0.58-6.77) |  |
| High Cholesterol | 0.19 (0.17-2.03) | 0.976 | 1.66 (1.3-9.24) | 0.464 |
| High triglycerides | 3.35 (2.54-17.24) | 0.310 | 0.62 (0.51-4.14) | 0.299 |
| High LDL | 5.22 (4.98-121.92) | 0.798 | 0.92 (0.85-12.66) | 0.968 |
| Low HDL | 2.12 (1.76-14.59) | 0.324 | 1.19 (1.08-14.21) | 0.100 |
| Poor controlled HbA1c (≥7.5) | 0.77 (0.66-5.94) | 0.557 | 0.67 (0.6-7.32) | 0.981 |

Estimated Odds Ratios (OR) and 95% Confidence Intervals (95% CI) for associations between cluster allocation (outcome; with the no/minimal NPS cluster as reference) and potential sociodemographic and health-related determinants in dementia free group. Reference group for the health-related factors: Male sex; Education Low (≤ 8 years); Unmarried for marital status; BMI normal for BMI categories; Absence of hypertension; Thyroid normal for thyroid functioning categories; HbA1c <7.5 for HbA1c %.

Abbreviations: NPS, neuropsychiatric symptoms; Dep, depression; Anx, anxiety; Apa, apathy; Irr, irritability; BMI, body mass index; ADL, activities of daily living; IADL, instrumental activities of daily living; CIRS-G, cumulative illness rating scale-geriatric; MMSE, mini mental state examination; LDL, low density lipoprotein; HDL, high density lipoprotein; HbA1C, hemoglobin A1c

**Supplementary Table** **7—**Demographic and clinical characteristics by clusters of neuropsychiatric symptoms in the GERICO cohort (n=1234) excluded depression and anxiety symptoms.

| **Characteristics** | **Cluster 0**  **No NPS**  **n = 561** | **Cluster 1**  **(Agi-Irr)**  **n = 137** | **Cluster 2**  **(Del-Agi-Irr)**  **n = 136** | **Cluster 3**  **(NightB)**  **n = 119** | **Cluster 4**  **(Apa)**  **n =281** | **P-value*** |
| --- | --- | --- | --- | --- | --- | --- |
| **Demographic** | | | | | | |
| Age. years | 79 (75.5-83) | 80 (75-84) | 81 (72.25-85) | 83 (80-86) | 80 (76-84) | 0.182 |
| Female sex | 373 (66.5) | 66 (48.2) | 89 (65.4) | 82 (68.9) | 183 (65.1) | <0.001 |
| Education  Low (≤ 8 years) | 400 (71.3) | 96 (70.1) | 103 (75.7) | 93 (78.2) | 202 (71.9) | 0.722 |
| High (> 8 years) | 161 (28.7) | 41 (29.9) | 33 (24.3) | 26 (21.8) | 79 (28.1) |  |
| Marital status  Unmarried | 151 (42.5) | 32 (34.4) | 51 (51.0) | 47 (61.8) | 85 (47.0) | 0.016 |
| Married or in a relationship | 204 (57.5) | 61 (65.6) | 49 (49.0) | 29 (38.2) | 96 (53.0) |  |
| BMI | 26.60 ± 4.44 | 26.41± 4.55 | 26.61± 4.61 | 26.81±5.06 | 26.38±4.34 | 0.984 |
| Underweight (<18.5) | 9 (1.6) | 4 (2.9) | 4 (2.9) | 3 (2.5) | 5 (1.8) | 0.985 |
| Normal (≥18.5–25) | 159 (28.3) | 43 (31.4) | 42 (30.9) | 29 (24.4) | 88 (31.3) |  |
| Overweight (≥25–30) | 178 (31.7) | 42 (30.7) | 48 (35.3) | 39 (32.8) | 93 (33.1) |  |
| Obesity (≥30) | 97 (17.3) | 26 (19.0) | 22 (16.1) | 93 (78.2) | 46 (16.4) |  |
| ADL score | 5 (5-6) | 5 (4-6) | 5 (4-6) | 4 (3-5) | 5 (4-6) | <0.001 |
| IADL score | 5 (2-7) | 3 (1-5) | 3 (1-5) | 1 (1-4) | 3 (1-5) | <0.001 |
| Depressive symptoms (GDS-15 score ≥5) | 214 (40.9) | 51 (42.1) | 73 (59.3) | 44 (48.9) | 141 (53.4) | <0.001 |
| Neuropsychiatric Inventory (NPI) Total score | 8 (4-14) | 27 (18-36) | 30 (23-39) | 30 (22-39) | 19 (13.5-26) | <0.001 |
| **Cognitive Status** | | | | | | |
| MMSE score | 23.15 ± 5.31 | 21.16 ± 6.84 | 21.26 ± 5.72 | 17.42 ± 6.19 | 21.39 ± 5.50 | <0.001 |
| Unimpaired | 43 (7.7) | 11 (8.0) | 4 (2.9) | 0 | 8 (2.8) | <0.001 |
| SCD | 55 (9.8) | 8 (5.8) | 6 (4.4) | 2 (1.7) | 8 (2.8) |  |
| MCI | 242 (43.1) | 43 (31.4) | 51 (37.5) | 14 (11.8) | 107 (38.1) |  |
| Dementia | 221 (39.4) | 75 (54.7) | 75 (55.1) | 103 (86.6) | 158 (56.2) |  |
| CDR=0 (UC and SCD) | 98 (17.5) | 19 (13.9) | 10 (7.4) | 2 (1.7) | 16 (5.7) | <0.001 |
| CDR= 0.5 (MCI) | 242 (43.1) | 43 (31.4) | 51 (37.5) | 14 (11.8) | 107 (38.1) |  |
| CDR= 1 (Mild dementia) | 163 (29.1) | 49 (35.8) | 44 (32.4) | 51 (42.9) | 108 (38.4) |  |
| CDR= 2 (Moderate dementia) | 52 (9.3) | 20 (14.6) | 29 (21.3) | 37 (31.1) | 44 (15.7) |  |
| CDR= 3 (Severe dementia) | 6 (1.1) | 6 (1.1) | 2 (1.5) | 15 (12.6) | 6 (2.1) |  |
| **Medical Conditions** | | | | | | |
| Cumulative Illness Rating Scale-Geriatric (CIRS_G) Total Score | 9 (6-12) | 9 (6-12) | 10 (7-15) | 10 (7-14) | 9 (6-12) | 0.039 |
| Comorbidity index | 1 (0-1) | 1 (0-2) | 1 (0-2) | 1 (0-2) | 1 (0-1) | <0.001 |
| Severity Index | 1.75 (1.43-2) | 1.81 (1.50-2.28) | 1.83 (1.57-2.14) | 2 (1.57-2.13) | 1.83 (1.50-2) | 0.523 |
| Number of medication | 5 (3-7) | 5 (4-7) | 6 (4-8) | 5 (4-8) | 6 (4-8) | 0.337 |
| Polypharmacy (>5 drugs) | 237 (55.1) | 71 (58.2) | 80 (73.4) | 67 (63.2) | 144 (63.7) | 0.021 |
| Stroke | 15 (2.7) | 6 (4.4) | 4 (2.9) | 8 (6.7) | 14 (5.0) | 0.266 |
| Myocardial infarction | 26 (4.6) | 5 (3.6) | 4 (2.9) | 2 (1.7) | 9 (3.2) | 0.437 |
| Diabetes | 138 (24.6) | 47 (34.3) | 31 (22.8) | 27 (22.7) | 65 (23.1) | 0.107 |
| Cancer | 75 (13.4) | 16 (11.7) | 20 (14.7) | 20 (16.8) | 47 (16.7) | 0.833 |
| Hyperlipidemia | 369 (65.8) | 97 (70.8) | 87 (64.0) | 84 (70.6) | 188 (66.9) | 0.833 |
| Hypertension | 386 (68.8) | 105 (76.6) | 109 (80.1) | 88 (73.9) | 206 (73.3) | 0.156 |
| Thyroid functioning  Thyroid_normal | 292 (52.0) | 91 (66.4) | 74 (54.4) | 76 (63.9) | 162 (57.7) | 0.977 |
| Hypothyroidism | 80 (14.3) | 18 (13.1) | 20 (14.7) | 20 (16.8) | 35 (12.5) |  |
| Hyperthyroidism | 43 (7.7) | 12 (8.8) | 9 (6.6) | 7 (5.9) | 16 (5.7) |  |
| **Blood biomarkers** | | | | | | |
| Total cholesterol (mg/dL)  High cholesterol | 193 (168-227)  229 (44.1) | 187.5 (161-222)  52 (39.4) | 192 (169-224)  53 (43.1) | 203 (169-236)  58 (51.8) | 191 (165-222)  109 (40.8) | 0.724  0.527 |
| HDL-c (mg/dL)  Low HDL | 55 (47-66)  52 (10.2) | 53 (43.5-64)  22 (17.1) | 54.5 (47-67.2)  16 (13.1) | 55 (45-67)  15 (13.5) | 56 (46-64.2)  32 (12.4) | 0.614  0.527 |
| LDL- (mg/dL)  High LDL | 113.9 (91.7-142)  59 (12.6) | 108 (84-134)  16 (13.9) | 115 (93.6-140)  12 (10.8) | 120 (96-150)  17 (16.2) | 112 (90.5-139.4)  27 (11.4) | 0.527  0.895 |
| Triglycerides (mg/dL)  High triglycerides | 108 (81-141)  101 (19.5) | 106.5 (81-155.5)  34 (25.8) | 102 (86-134)  23 (19.0) | 108 (81-137)  22 (19.8) | 106 (81-146.2)  62 (23.3) | 0.984  0.702 |
| Glucose (mg/dL) | 100 (91-114) | 103 (90-120) | 101 (91-116.5) | 99 (90-117) | 99 (90.2-113.7) | 0.984 |
| HbA1C % | 6.3 (5.7-7) | 6.7 (5.7-7.4) | 6.3 (5.8-7.1) | 6.1 (5.7-7) | 6.3 (5.6-6.9) | 0.984 |
| Poor controlled (≥7.5) | 20 (18.5) | 6 (24.0) | 6 (22.2) | 4 (18.2) | 8 (14.5) | 0.984 |
| Vit D (ng/mL) | 31.2 (20-43.5) | 27.8 (18.2-38.1) | 30.8 (17.8-60.2) | 24.7 (18.2-35.5) | 32.3 (23.6-40.7) | 0.883 |
| Vit B12 (pg/ml) | 242 (182.5-334) | 220 (177-308) | 222 (174.5-322) | 233 (175-354) | 239 (190-318) | 0.783 |
| TSH (μUI/ml) | 1.74 (1.13-2.5) | 1.79 (1.36-2.79) | 1.51 (0.99-2.38) | 1.57 (0.92-2.34) | 1.63 (1.06-2.40) | 0.150 |

Data presented as number (proportion %), means ± standard deviations, or median (25th–75th percentile) with p-values calculated using Chi-square, one-way ANOVA, or Kruskal-Wallis test, respectively.

Abbreviations: NPS, neuropsychiatric symptoms; Del, delusion; Agi, agitation; Apa, apathy; Irr, irritability; NightB, nightime behavioral disturbances; BMI, body mass index; ADL, activities of daily living; IADL, instrumental activities of daily living; MMSE, mini mental state examination; SCD, subjective cognitive decline; MCI, mild cognitive impairment; CDR, clinical dementia rating; NPI, neuropsychiatric inventory; CIRS_G, cumulative illness rating scale-geriatric; LDL-c, low density lipoprotein; HDL-c, high density lipoprotein; HbA1C, hemoglobin A1c; TSH. thyroid-stimulating hormone.

Missing data: 429 for Marital Status; 235 for BMI; 113 for GDS; 241 for polypharmacy; 140 for CIRS_G; 140 for CIRS_G_Comorbidity Index; 140 for CIRS_G_Severity Index; 241 for Number of medications; 136 for Stroke; 136 for Myocardial infarction; 136 for Cancer; 279 for Thyroid; 81 for Cholesterol; 199 LDL-c; 103 for HDL-c; 87 for Triglycerides; 85 for Glucose; 997 for HbA1c; 962 for Vitamin D; 95 for Vitamin B12; 93 for TSH.

* P-values are reported after FDR adjustment for multiple comparisons

**Supplementary Table 8**—Associations between cluster allocation and potential sociodemographic and health-related determinants in overall sample excluding anxiety and depression.

|  | **Cluster 1 (n =137)** | | **Cluster 2 (n =136)** | | **Cluster 3 (n =119)** | | **Cluster 4 (n = 281)** | |
| --- | --- | --- | --- | --- | --- | --- | --- | --- |
|  | **(Agi-Irr)** | | **(Del-Agi-Irr)** | | **(NightB)** | | **(Apa)** | |
|  | **OR (95%CI)** | **p-values** | **OR (95%CI)** | **p-values** | **OR (95%CI)** | **p-values** | **OR (95%CI)** | **p-values** |
| Age, year | 1 (0.04-2.04) | 0.911 | 1.04 (0.04-2.12) | 0.002 | 1.12 (0.05-2.29) | <0.001 | 1.02 (0.03-2.07) | 0.039 |
| Female sex | 0.41 (0.16-1.09) | <0.001 | 0.77 (0.31-2.05) | 0.817 | 1.23 (0.57-3.53) | 0.611 | 0.76 (0.25-1.89) | 0.693 |
| Education, High (> 8 years) | 1.03 (0.41-2.74) | 0.776 | 0.87 (0.35-2.33) | 0.301 | 0.91 (0.42-2.58) | 0.128 | 0.97 (0.32-2.43) | 0.856 |
| Marital Status. Married or in a relationship | 1.04 (0.43-2.81) | 0.156 | 0.74 (0.29-1.95) | 0.132 | 0.67 (0.29-1.85) | 0.002 | 0.81 (0.26-2.01) | 0.329 |
| BMI underweight | 1.16 (0.88-5.9) | 0.901 | 1.64 (1.25-8.46) | 0.974 | 1.11 (0.91-7.34) | 0.762 | 1.14 (0.79-4.88) | 0.643 |
| BMI overweight | 0.92 (0.39-2.5) |  | 1.02 (0.43-2.8) |  | 1.68 (0.79-4.86) |  | 1.03 (0.36-2.62) |  |
| BMI obese | 1.07 (0.5-3.07) |  | 0.74 (0.37-2.2) |  | 1.4 (0.73-4.33) |  | 0.8 (0.33-2.18) |  |
| ADL score | 1.06 (0.22-2.4) | 0.062 | 0.83 (0.17-1.88) | <0.001 | 0.84 (0.18-1.92) | <0.001 | 1.08 (0.19-2.39) | 0.013 |
| IADL score | 0.82 (0.1-1.75) | <0.001 | 0.93 (0.11-1.99) | <0.001 | 0.67 (0.1-1.46) | <0.001 | 0.8 (0.08-1.69) | <0.001 |
| Hypertension | 0.56 (0.27-1.63) | 0.071 | 1.06 (0.59-3.43) | 0.008 | 0.76 (0.41-2.42) | 0.268 | 0.81 (0.35-2.23) | 0.177 |
| Number of medications | 1.03 (0.1-2.16) | 0.556 | 1.05 (0.1-2.21) | <0.001 | 0.96 (0.1-2.03) | 0.118 | 1.04 (0.08-2.16) | 0.031 |
| CIRS_G (total score) | 1 (0.05-2.06) | 0.188 | 1.07 (0.05-2.19) | <0.001 | 1.01 (0.06-2.09) | <0.001 | 1 (0.04-2.05) | 0.123 |
| MMSE score | 0.94 (0.03-1.91) | <0.001 | 0.94 (0.03-1.91) | <0.001 | 0.86 (0.03-1.74) | 0.003 | 0.94 (0.02-1.91) | <0.001 |
| Hypothyroidism | 0.76 (0.36-2.19) | 0.351 | 0.95 (0.46-2.78) | 0.265 | 1.05 (0.48-2.98) | 0.423 | 0.94 (0.36-2.48) | 0.877 |
| Hyperthyroidism | 0.69 (0.37-2.18) |  | 0.89 (0.48-2.82) |  | 0.43 (0.26-1.55) |  | 0.61 (0.29-1.77) |  |
| High Cholesterol | 0.76 (0.31-2.04) | 0.328 | 0.94 (0.39-2.53) | 0.835 | 1.18 (0.49-3.18) | 0.140 | 0.71 (0.24-1.79) | 0.376 |
| High triglycerides | 1.21 (0.5-3.27) | 0.116 | 1.12 (0.5-3.14) | 0.895 | 1.02 (0.44-2.83) | 0.945 | 1.59 (0.55-4.02) | 0.218 |
| High LDL | 1.64 (0.85-5.05) | 0.708 | 0.95 (0.54-3.14) | 0.604 | 1.63 (0.82-4.91) | 0.328 | 1.4 (0.64-3.98) | 0.656 |
| Low HDL | 1.68 (0.76-4.76) | 0.034 | 0.94 (0.5-2.95) | 0.347 | 1.27 (0.64-3.84) | 0.304 | 1.19 (0.49-3.19) | 0.350 |

Estimated Odds Ratios (OR) and 95% Confidence Intervals (95% CI) for associations between cluster allocation (outcome; with the no/minimal NPS cluster as reference) and potential sociodemographic and health-related determinants in overall sample excluding anxiety and depression. Reference group for the health-related factors: Male sex; Education Low (≤ 8 years); Unmarried for marital status; BMI normal for BMI categories; Absence of hypertension; Thyroid normal for thyroid functioning categories. HbA1c was excluded from the sub-analysis models owing to the larger amount of missing data.

Abbreviations: Del, delusion; Agi, agitation; Apa, apathy; Irr, irritability; NightB, nighttime behavioral disturbances; BMI, body mass index; ADL, activities of daily living; IADL, instrumental activities of daily living; CIRS-G, cumulative illness rating scale-geriatric; MMSE, mini mental state examination; LDL, low density lipoprotein; HDL, high density lipoprotein; HbA1C, hemoglobin A1c.

**Supplementary Table 9**—Associations between cluster allocation and potential sociodemographic and health-related determinants in dementia sample excluding anxiety and depression.

|  | **Cluster 1 (n =264)** | | **Cluster 2 (n = 85)** | | **Cluster 3 (n = 92)** | |
| --- | --- | --- | --- | --- | --- | --- |
|  | **(Apa)** | | **(Del)** | | **(Agi-Apa-Irr)** | |
|  | **OR (95%CI)** | **p-values** | **OR (95%CI)** | **p-values** | **OR (95%CI)** | **p-values** |
| Age, year | 1.01 (0.04-2.06) | 0.781 | 1.07 (0.06-2.21) | 0.257 | 1.05 (0.06-2.17) | 0.001 |
| Female sex | 1.46 (0.62-4) | 0.309 | 0.47 (0.24-1.45) | 0.020 | 1.88 (1.08-6.27) | 0.368 |
| Education, High (> 8 years) | 1.55 (0.72-4.44) | 0.992 | 2.08 (1.12-6.6) | 0.562 | 1.38 (0.82-4.78) | 0.548 |
| Marital Status. Married or in a relationship | 0.88 (0.35-2.33) | 0.314 | 0.94 (0.49-2.89) | 0.615 | 0.58 (0.3-1.79) | 0.011 |
| BMI underweight | 0.98 (0.78-5.67) | 0.046 | 2.89 (2.32-17.55) | 0.074 | 1.76 (1.5-13.79) | 0.338 |
| BMI overweight | 1.28 (0.53-3.45) |  | 1.55 (0.8-4.74) |  | 1.93 (1.03-6.09) |  |
| BMI obese | 1.05 (0.49-3.01) |  | 0.82 (0.5-2.95) |  | 1.92 (1.11-6.5) |  |
| ADL score | 1.02 (0.19-2.27) | 0.115 | 1.16 (0.29-2.71) | 0.914 | 0.85 (0.2-1.96) | <0.001 |
| IADL score | 1.1 (0.15-2.37) | 0.071 | 0.87 (0.16-1.94) | 0.115 | 0.86 (0.18-1.93) | 0.002 |
| Hypertension | 0.6 (0.31-1.83) | 0.171 | 0.45 (0.27-1.56) | 0.186 | 0.57 (0.36-2.11) | 0.239 |
| Number of medications | 1 (0.09-2.1) | 0.040 | 0.96 (0.12-2.05) | 0.176 | 0.95 (0.12-2.03) | 0.425 |
| CIRS_G (total score) | 0.98 (0.05-2) | 0.145 | 1.02 (0.07-2.11) | 0.915 | 1.02 (0.07-2.1) | 0.895 |
| MMSE score | 1.01 (0.04-2.06) | 0.659 | 0.98 (0.05-2.01) | 0.452 | 0.92 (0.04-1.89) | <0.001 |
| Hypothyroidism | 0.86 (0.41-2.48) | 0.946 | 1.4 (0.75-4.41) | 0.184 | 0.8 (0.46-2.67) | 0.305 |
| Hyperthyroidism | 2.52 (1.49-8.69) |  | 1.23 (0.89-5.69) |  | 1.29 (0.9-5.59) |  |
| High Cholesterol | 1.1 (0.45-2.96) | 0.147 | 0.66 (0.35-2.07) | 0.644 | 1.64 (0.8-4.85) | 0.019 |
| High triglycerides | 0.49 (0.21-1.34) | 0.047 | 0.71 (0.36-2.14) | 0.324 | 0.65 (0.32-1.93) | 0.312 |
| High LDL | 1.07 (0.59-3.44) | 0.479 | 2.6 (1.64-9.66) | 0.115 | 0.84 (0.53-3.12) | 0.696 |
| Low HDL | 0.5 (0.24-1.45) | 0.026 | 0.91 (0.49-2.89) | 0.828 | 0.88 (0.49-2.86) | 0.641 |

Estimated Odds Ratios (OR) and 95% Confidence Intervals (95% CI) for associations between cluster allocation (outcome; with the no/minimal NPS cluster as reference) and potential sociodemographic and health-related determinants in dementia sample excluding anxiety and depression. Reference group for the health-related factors: Male sex; Education Low (≤ 8 years); Unmarried for marital status; BMI normal for BMI categories; Absence of hypertension; Thyroid normal for thyroid functioning categories. HbA1c was excluded from the sub-analysis models owing to the larger amount of missing data.

Abbreviations: Del, delusion; Agi, agitation; Apa, apathy; Irr, irritability; BMI, body mass index; ADL, activities of daily living; IADL, instrumental activities of daily living; CIRS-G, cumulative illness rating scale-geriatric; MMSE, mini mental state examination; LDL, low density lipoprotein; HDL, high density lipoprotein; HbA1C, hemoglobin A1c.

**Supplementary Table 10**—Associations between cluster allocation and potential sociodemographic and health-related determinants in dementia free sample excluding anxiety and depression.

|  | **Cluster 1 (n = 368)** | | **Cluster 2 (n = 91)** | |
| --- | --- | --- | --- | --- |
|  | **(Apa)** | | **(Agi-Irr)** | |
|  | **OR (95%CI)** | **p-values** | **OR (95%CI)** | **p-values** |
| Age, year | 0.98 (0.04-1.99) | 0.135 | 0.95 (0.05-1.95) | 0.139 |
| Female sex | 1.07 (0.43-2.85) | 0.469 | 0.53 (0.27-1.61) | 0.036 |
| Education, High (> 8 years) | 0.83 (0.32-2.19) | 0.877 | 0.94 (0.47-2.82) | 0.589 |
| Marital Status. Married or in a relationship | 1.67 (0.66-4.44) | 0.020 | 0.85 (0.44-2.60) | 0.549 |
| BMI underweight | 0.66 (0.54-4.42) | 0.230 | 0.83 (0.77-11.23) | 0.136 |
| BMI overweight | 0.73 (0.34-2.11) |  | 1.22 (0.72-4.17) |  |
| BMI obese | 1.2 (0.66-3.86) |  | 2.12 (1.36-8.04) |  |
| ADL score | 0.96 (0.29-2.33) | 0.094 | 0.71 (0.25-1.81) | 0.308 |
| IADL score | 1.27 (0.17-2.74) | <0.001 | 1.23 (0.21-2.71) | 0.431 |
| Hypertension | 1.86 (1.04-6.06) | 0.673 | 1.33 (0.88-5.24) | 0.115 |
| Number of medications | 0.94 (0.1-2.00) | 0.071 | 1.01 (0.13-2.18) | 0.733 |
| CIRS_G (total score) | 0.99 (0.07-2.05) | 0.118 | 0.98 (0.08-2.05) | 0.492 |
| MMSE score | 1.07 (0.06-2.2) | 0.034 | 1.06 (0.09-2.2) | 0.187 |
| Hypothyroidism | 0.98 (0.47-2.87) | 0.737 | 0.41 (0.26-1.54) | 0.230 |
| Hyperthyroidism | 0.76 (0.42-2.44) |  | 0.82 (0.53-3.12) |  |
| High Cholesterol | 1.98 (0.91-5.66) | 0.880 | 1.49 (0.83-4.84) | 0.367 |
| High triglycerides | 0.92 (0.43-2.65) | 0.568 | 0.97 (0.53-3.13) | 0.141 |
| High LDL | 0.36 (0.20-1.19) | 0.607 | 0.89 (0.58-3.43) | 0.830 |
| Low HDL | 1.26 (0.73-4.27) | 0.626 | 3.07 (1.93-11.3) | 0.022 |

Estimated Odds Ratios (OR) and 95% Confidence Intervals (95% CI) for associations between cluster allocation (outcome; with the no/minimal NPS cluster as reference) and potential sociodemographic and health-related determinants in dementia free sample excluding anxiety and depression. Reference group for the health-related factors: Male sex; Education Low (≤ 8 years); Unmarried for marital status; BMI normal for BMI categories; Absence of hypertension; Thyroid normal for thyroid functioning categories. HbA1c was excluded from the sub-analysis models owing to the larger amount of missing data.

Abbreviations: Agi, agitation; Apa, apathy; Irr, irritability; BMI, body mass index; ADL, activities of daily living; IADL, instrumental activities of daily living; CIRS-G, cumulative illness rating scale-geriatric; MMSE, mini mental state examination; LDL, low density lipoprotein; HDL, high density lipoprotein; HbA1C, hemoglobin A1c.

**Supplementary Figure 1—**Optimal number of clusters for the overall sample, dementia group, and dementia-free group.

**
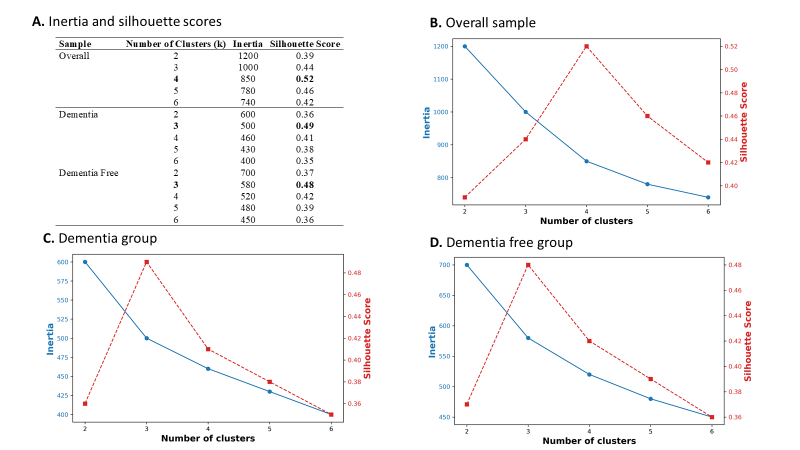
**

Panel A shows the table summarizing inertia and silhouette scores for k = 2–6 across the three samples. Panels B–D display elbow plots and silhouette scores for the overall sample (B), dementia group (C), and dementia-free group (D). For each subgroup, inertia (blue solid line, left y-axis) and silhouette score (red dashed line, right y-axis) are plotted as a function of the number of clusters (k). Lower inertia values indicate greater within-cluster compactness, whereas higher silhouette scores reflect better-defined and more separated clusters. The optimal number of clusters was four for the overall sample and three for both dementia and dementia-free subgroups.

**Supplementary Figure 2—**Agreement between Euclidean k-means and alternative distance-clustering solutions.
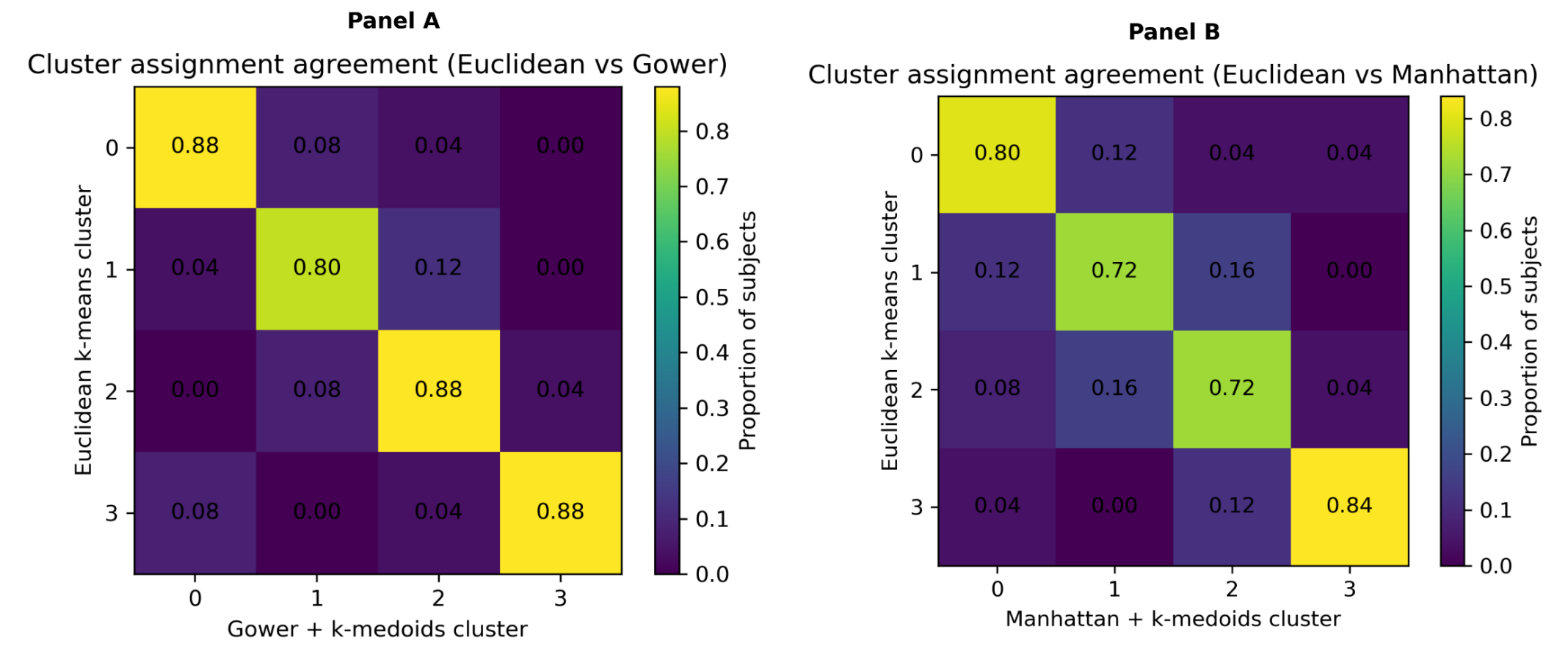


Panel A (left) shows the agreement between cluster assignments obtained using Euclidean k-means clustering and Gower distance-based k-medoids clustering. Panel B (right) shows the agreement between Euclidean k-means and Manhattan-distance k-medoids clustering. The heatmaps display the proportion of participants assigned to each cluster by Euclidean k-means (rows) versus the alternative distance-based method (columns). Diagonal cells indicate concordant cluster assignments, whereas off‑diagonal cells represent reassignments. Overall, the higher diagonal concordance—particularly for the Gower distance—demonstrates that the identified cluster structure is robust to distance metrics that account for the ordinal nature of the symptom scores.

**Supplementary Figure 3—**Sociodemographic, clinical and systemic biomarkers feature of the NPS clusters in the overall sample excluding depression and anxiety.


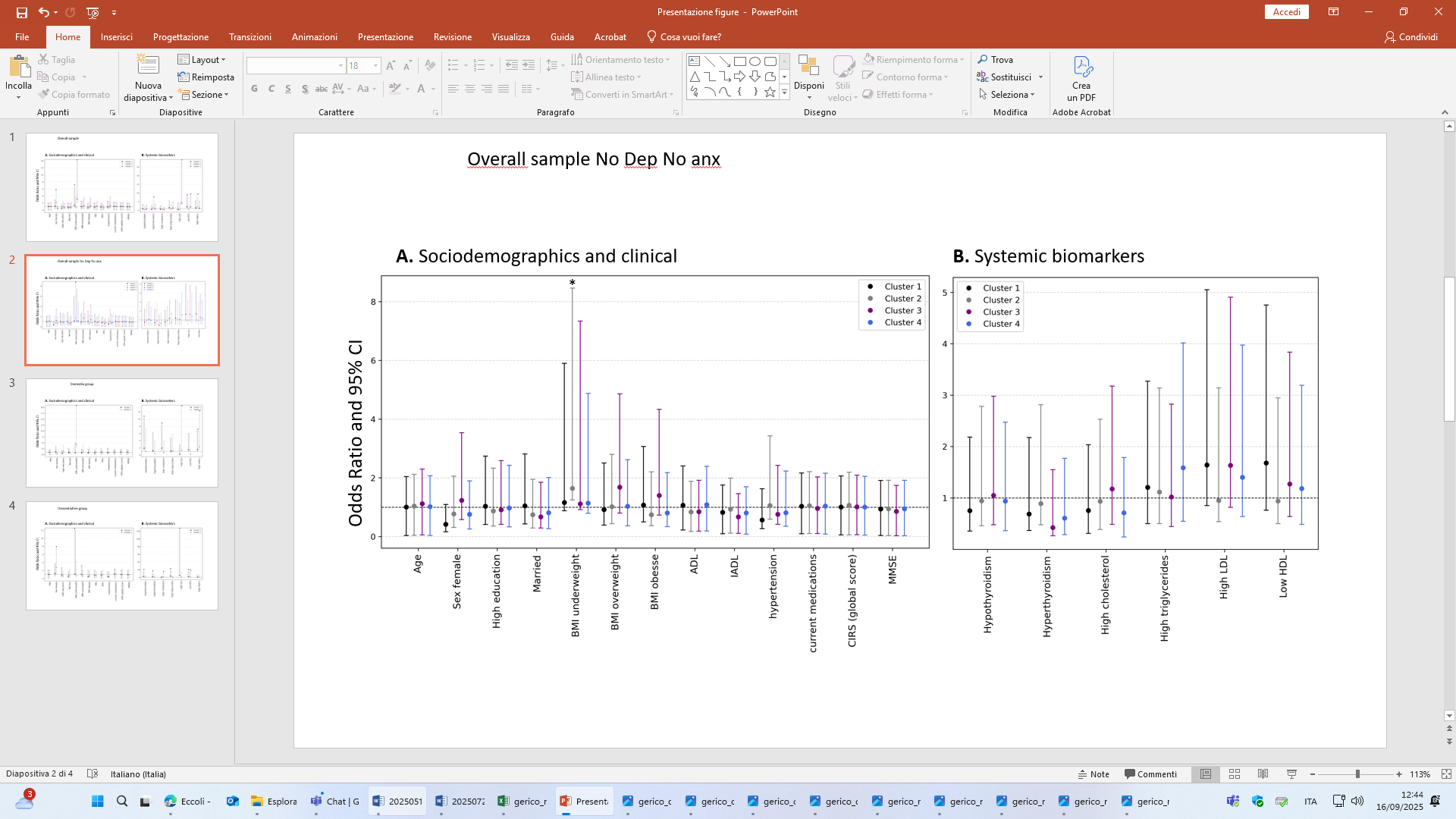


The figure displays multinomial logistic regression models’ Odds Ratios and 95% CIs for associations between cluster allocation (outcome; with the no/minimal NPS cluster as the reference) and Sociodemographic and clinical features (panel A) and systemic biomarkers (panel B) in overall sample excluding anxiety and depression. Cluster 1 “Agitation-Irritability” (circle black); Cluster 2 “Delusion-Agitation-Irritability” (circle grey); Cluster 3 “Nighttime behaviors” (circle purple); Cluster 4 “Apathy” (circle blue) vs. Cluster 0 “no/minimal NPS” (reference). * Statistically significant odds ratios. Abbreviations: BMI, body mass index; ADL, activities of daily living; IADL, instrumental activities of daily living; CIRS-G, cumulative illness rating scale-geriatric; MMSE, mini mental state examination; LDL, low density lipoprotein; HDL, high density lipoprotein.

**Supplementary Figure 4—**Sociodemographic, clinical and systemic biomarkers feature of the NPS clusters in the dementia group excluding depression and anxiety.


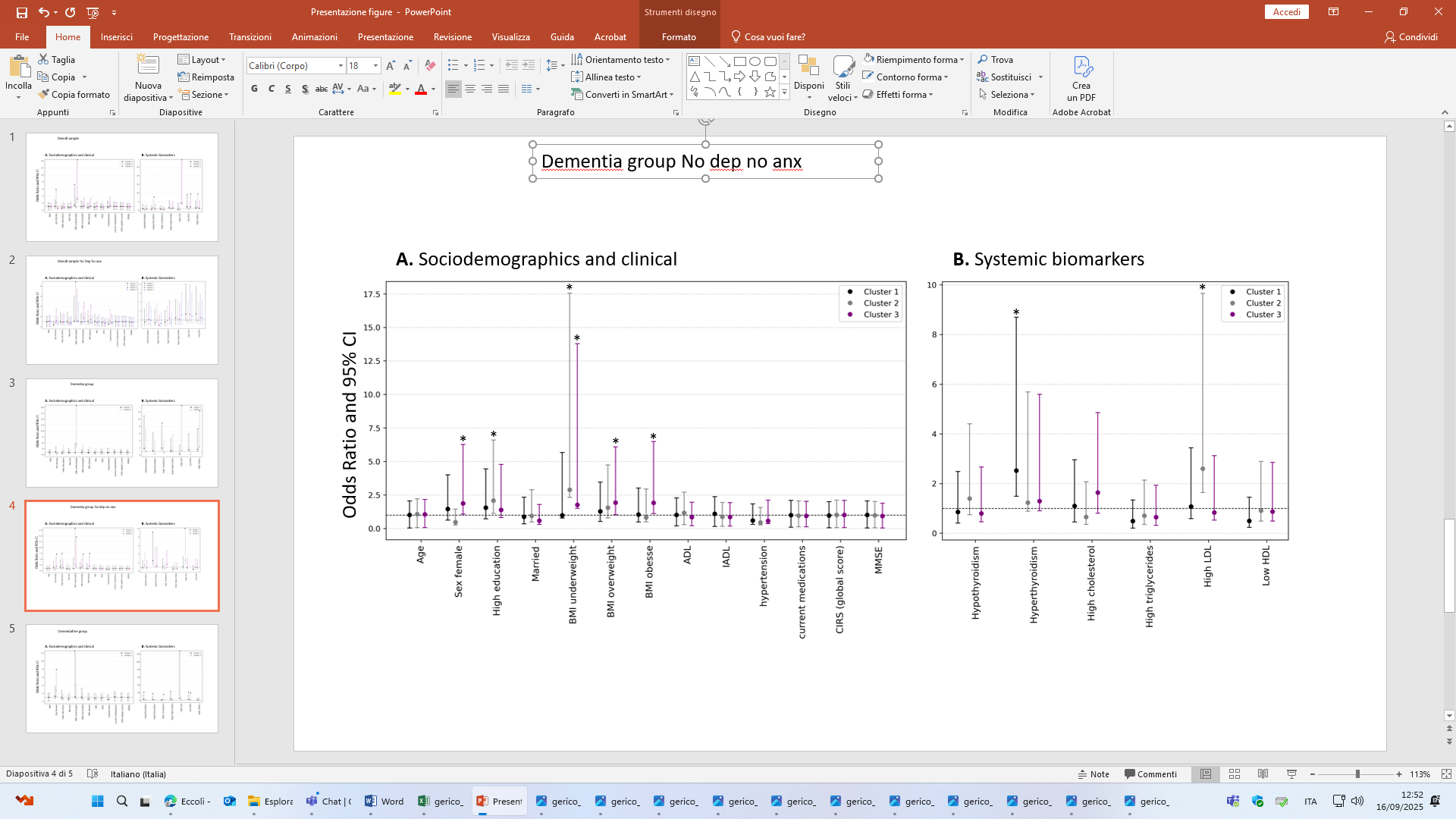


The figure displays multinomial logistic regression models’ Odds Ratios and 95% CIs for associations between cluster allocation (outcome; with the no/minimal NPS cluster as the reference) and Sociodemographic and clinical features (panel A) and systemic biomarkers (panel B) in dementia group excluding anxiety and depression. Cluster 1 “Apathy” (circle black); Cluster 2 “Delusion” (circle grey); Cluster 3 “Agitation-Apathy-Irritability-Nighttime behaviors” (circle purple) vs. Cluster 0 “no/minimal NPS” (reference). * Statistically significant odds ratios.

Abbreviations: BMI, body mass index; ADL, activities of daily living; IADL, instrumental activities of daily living; CIRS-G, cumulative illness rating scale-geriatric; MMSE, mini mental state examination; LDL, low density lipoprotein; HDL, high density lipoprotein.

**Supplementary Figure 5—**Sociodemographic, clinical and systemic biomarkers feature of the NPS clusters in the dementia free group excluding depression and anxiety.


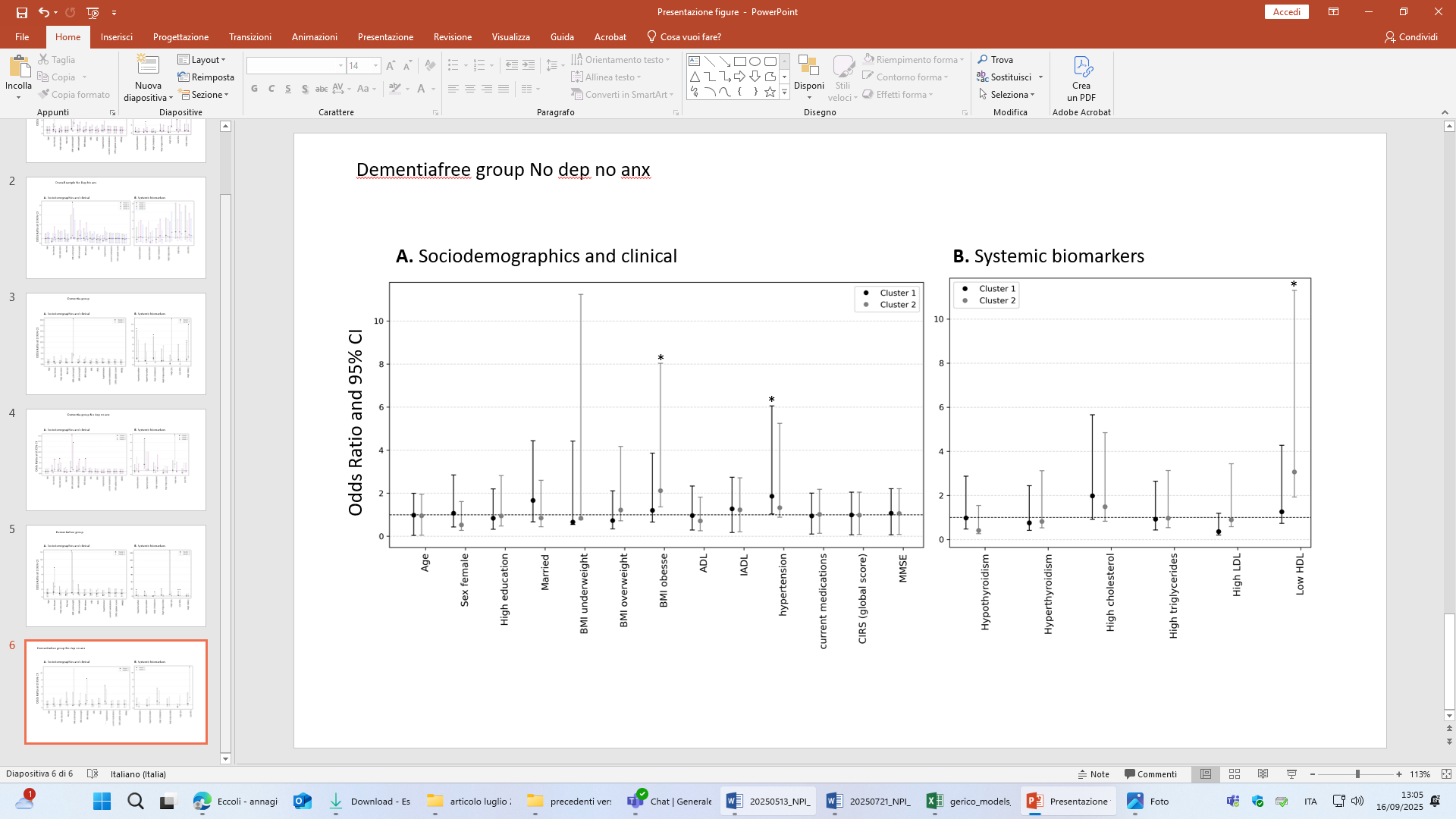


The figure displays multinomial logistic regression models’ Odds Ratios and 95% CIs for associations between cluster allocation (outcome; with the no/minimal NPS cluster as the reference) and Sociodemographic and clinical features (panel A) and systemic biomarkers (panel B) in dementia free group excluding anxiety and depression. Cluster 1 “Apathy” (circle black); Cluster 2 “Agitation-Irritability” (circle grey); vs. Cluster 0 “no/minimal NPS” (reference). * Statistically significant odds ratios. Abbreviations: BMI, body mass index; ADL, activities of daily living; IADL, instrumental activities of daily living; CIRS-G, cumulative illness rating scale-geriatric; MMSE, mini mental state examination; LDL, low density lipoprotein; HDL, high density lipoprotein.
